# Immunogenicity and protective efficacy of single-dose adenoviral vaccines expressing Crimean-Congo haemorrhagic fever virus GP38, Gc, or nucleoprotein antigens in mice: a preclinical study

**DOI:** 10.1016/j.ebiom.2026.106531

**Published:** 2026-10-21

**Authors:** Jack E. Saunders, Ciaran Gilbride, Alex Sampson, Stuart Dowall, Emma Kennedy, Sarah Webster, Susan Fotheringham, Stephen Findlay–Wilson, Fahad Al-Dainy, Susana Camara, Khiyam Hussain, Ines Ruedas-Torres, Francisco J. Salguero, Roger Hewson, Sarah C. Gilbert, Sagida Bibi, Daniel Wright, Sandra Belij-Rammerstorfer, Teresa Lambe

**Affiliations:** a Oxford Vaccine Group (OVG), Department of Paediatrics, University of Oxford, Oxford, UK; b UK Health Security Agency (UKHSA), Porton Down, Salisbury, Wiltshire, UK; c Pandemic Sciences Institute (PSI), Nuffield Department of Medicine, University of Oxford, Oxford, UK; d Chinese Academy of Medical Science (CAMS) Oxford Institute, University of Oxford, Oxford, UK

**Keywords:** Crimean-Congo haemorrhagic fever, ChAdOx vaccine, Glycoprotein precursor (GPC), Nucleoprotein (NP), IFNAR−/− mouse model, Protective immunity

## Abstract

**Background:**

Crimean-Congo haemorrhagic fever virus (CCHFV) is a highly pathogenic, tick-borne orthonairovirus responsible for severe human disease, yet no approved vaccines or therapeutics exist. The glycoprotein precursor (GPC), including its GP38, Gn, and Gc components, and the nucleoprotein (NP) have been explored as vaccine targets with varying success. A chimpanzee adenoviral vector (ChAdOx) vaccine encoding full-length GPC previously conferred complete protection in mice, supporting Phase I evaluation. However, the essential protective components and mechanisms of vaccine-induced immunity remain unclear.

**Methods:**

We evaluated ChAdOx2 vaccines encoding individual GPC subunits (GP38, Gn, or Gc), full-length GPC, or NP in a lethal interferon receptor-deficient mouse model. Protective efficacy and immune responses were assessed to define protective immunogens and mechanisms.

**Findings:**

Single-dose vaccination with ChAdOx2-GP38 or ChAdOx2-Gc conferred complete survival (6/6 per group; both *p* = 0.0005 vs PBS), with minimal histopathology and viral RNA burden at the 21-day study endpoint, comparable to full-length GPC (6/6; *p* = 0.0005 vs PBS). ChAdOx2-NP likewise conferred complete survival (6/6; *p* = 0.0008 vs PBS) and elicited robust NP-specific immune responses. In contrast, ChAdOx2-Gn conferred partial survival (2/6; *p* = 0.0101 vs PBS), while low-dose GPC vaccination provided limited protection (1/6; *p* = 0.0617 vs PBS) with impaired IgG responses while preserving T-cell responses. Immunological analyses showed that GP38, Gc, and full-length GPC constructs induced robust antibody responses with similar Fcγ receptor engagement and limited neutralising activity.

**Interpretation:**

These findings identify GP38 and Gc as individually protective immunogens while reinforcing GPC and NP as effective vaccine targets. Together, these data support roles for non-neutralising antibodies and T-cell responses in protection against CCHFV and continued development of ChAdOx-vectored vaccines for clinical use.

**Funding:**

This research was supported by the Biotechnology and Biological Sciences Research Council.

**Research in context**

**Evidence before this study**

Crimean-Congo haemorrhagic fever virus (CCHFV) is a WHO-priority pathogen that causes severe human disease with case fatality rates of up to 30% and is endemic across regions of Africa, Asia, the Middle East, and Europe. We searched PubMed and Google Scholar from database inception up to 24 August 2026 using combinations of the terms “Crimean-Congo haemorrhagic fever”, “CCHFV”, “vaccine”, “protection”, “glycoprotein precursor”, “GPC”, “GP38”, “Gn”, “Gc”, “nucleoprotein”, and “NP”. We considered peer-reviewed preclinical studies evaluating the immunogenicity or protective efficacy of CCHFV vaccine candidates alongside available information on candidates in clinical development. No language restrictions were applied, and no formal risk-of-bias assessment was undertaken. No CCHF vaccines are licenced to contemporary regulatory standards, although several vaccine approaches using the full-length glycoprotein precursor (GPC) have demonstrated protection in preclinical models, including our previous study using a chimpanzee adenovirus platform (ChAdOx). Individual GPC components (GP38, Gn, and Gc), as well as nucleoprotein (NP), have also been evaluated for immunogenicity and protective efficacy as vaccine antigens. However, the immune pathways underlying GPC- or NP-mediated protection and the contribution of individual GPC components remain unclear. Defining the individual protective antigens and elucidating the mechanisms by which they elicit immunity are crucial for understanding protective responses and guiding the design of scalable, targeted vaccines.

**Added value of this study**

Building on our recent study demonstrating that a ChAdOx2-vectored vaccine encoding the full-length CCHFV GPC confers protective immunity, we sought to define the GPC-derived antigen targets sufficient to confer protective immunity. To define individually protective antigens, we engineered ChAdOx2 constructs expressing GP38, Gn, or Gc. Vaccines encoding GP38 or Gc alone conferred complete protection against lethal disease comparable to that observed with full-length GPC in an interferon receptor-deficient mouse model, whereas Gn vaccination provided incomplete protection. A 100-fold dose reduction of ChAdOx2-GPC substantially reduced protection, coinciding with loss of antibody responses despite preserved T-cell immunity. Detailed analyses of antibody binding and functionality indicated that non-neutralising responses against Gc and GP38 are likely to contribute to protection, although modest neutralising activity was detected in the Gc-vaccinated group. In addition, we evaluated a ChAdOx2 vaccine encoding NP and found it also conferred complete single-dose protection, driven by T-cell and non-neutralising antibody responses.

**Implications of all the available evidence**

Our findings identify GP38 and Gc as standalone protective GPC antigens for the ChAdOx2 platform, while reinforcing NP and full-length GPC as effective vaccine targets. Protective immunity was associated with humoural responses, with non-neutralising antibodies likely contributing to protection following full-length GPC immunisation, and T-cell responses providing additional protection, particularly in the context of NP. These insights support streamlined antigen design and suggest that combining NP with Gc or GP38 may enhance vaccine breadth across diverse CCHFV strains and improve durability. Together with the ongoing clinical evaluation of ChAdOx2-GPC, this work advances the rational development of CCHFV vaccines for outbreak response and pandemic preparedness.

## Introduction

Crimean-Congo haemorrhagic fever (CCHF) is a severe zoonotic, tick-borne viral disease in humans that has a mean case fatality rate around 30% or higher.^1^ The causative agent, CCHF virus (CCHFV), is a member of the genus *Orthonairovirus*, family *Nairoviridae*, within the order *Hareavirales* and class *Bunyaviricetes*, and has the potential to cause severe epidemics.^2^, ^3^ CCHFV is maintained asymptomatically in animal hosts and transmitted to humans primarily through bites from *Hyalomma* ticks, with additional transmission occurring via contact with blood, bodily fluids, or tissues from infected animals or humans.^4^, ^5^, ^6^, ^7^ Endemic throughout Africa, the Middle East, and Asia, CCHFV is expanding into Europe as populations of ticks of the *Hyalomma* genus shift with climate and land-use change.^8^, ^9^ Autochthonous cases have already been recorded in Spain and Bulgaria, in addition to recent outbreaks in Iraq (2022–2023) and Afghanistan (2023).^10^, ^11^, ^12^ In the absence of licenced medical interventions, the development of safe and effective CCHFV vaccines is critical to protect populations in endemic regions and reduce the risk of future outbreaks.

Vaccine approaches targeting the CCHFV nucleoprotein (NP) or glycoprotein precursor (GPC) have had varying platform-specific success, from full protection to limited efficacy in rodent and non-human primate models, and the immune correlates of protection remain unclear.^13^, ^14^, ^15^, ^16^, ^17^, ^18^, ^19^ Candidates encoding full-length GPC generally induce both cellular and humoural immunity and can protect in mouse models, though the mechanisms of protection induced by full-length GPC and the roles of its individual components remain poorly defined.^15^, ^20^, ^21^, ^22^ To dissect these contributions, recent studies have focused on truncated GPC-derived constructs; however, vaccines based solely on Gn or Gc are generally less effective unless combined with additional GPC components or NP.^23^, ^24^ Notably the GPC-derived GP38 is emerging as a promising standalone immunogen, improving survival in murine models and underscoring its potential role in mediating protection.^25^, ^26^, ^27^, ^28^ Alternatively, NP is also a viable vaccine target that can induce antibody and T-cell responses; the high conservation of NP among CCHFV genotypes makes it an attractive component for CCHFV vaccine design.^29^, ^30^, ^31^, ^32^, ^33^

Chimpanzee adenoviral (ChAd) vectors combine strong immunogenicity, safety, scalable low-cost production, and have been deployed globally for emergency vaccination during the COVID-19 pandemic.^34^, ^35^, ^36^, ^37^, ^38^ A ChAdOx2 candidate encoding full-length CCHFV GPC is in Phase I clinical trials (ISRCTN12351734), and recently provided complete single-dose protection in mice, inducing high titres of anti-Gc antibodies and GPC-specific T cells.^21^ However, the full GPC is near the adenoviral insert size limit and includes regions that may be dispensable for protection, with practical implications for antigen optimisation and vector design.^1^, ^39^ Using the ChAdOx2 platform to assess truncated or smaller CCHFV antigens could better define the critical antigen targets for protective immunity, facilitate identification of correlates of protection, and enhance vaccine manufacturability.

In this study, we undertook a comprehensive evaluation of engineered ChAdOx2 vaccines expressing individual GPC subunits (Gn, Gc, and GP38) as well as NP, alongside full-length GPC. Vaccine efficacy was evaluated in the interferon α/β receptor-deficient A129 mouse model of CCHF, in parallel with detailed immunological profiling, including antibody neutralising ability, IgG subclass distribution, Fcγ receptor binding to antigen-specific IgG, and cellular cytokine secretion. These studies revealed NP, GP38, and Gc as individually protective antigens, with GP38 and Gc acting as dominant contributors to the protective efficacy of ChAdOx2-expressed full-length GPC through non-neutralising antibody responses. Together, these evaluations highlight feasible antigen targets and provide mechanistic insight into vaccine-mediated protection, advancing the development of CCHFV vaccines.

## Methods

### Ethics statement

All animal procedures were undertaken at the UK Health Security Agency (UKHSA) Porton Down facility, UK, and were approved by the UKHSA Ethical Review Process and the Home Office, UK, via project licence number PP3877532. Work was performed in accordance with the Animals (Scientific Procedures) Act 1986 and the Home Office (UK) Code of Practice for the Housing and Care of Animals Used in Scientific Procedures (1989). Protocols and experiments were designed according to ARRIVE guidelines. Mice were housed in groups of three per cage prior to challenge and maintained under negative pressure conditions; following CCHFV challenge, animals were transferred to flexible isolators under climate-controlled conditions in a Biocontainment Level-4 facility (CL-4). Food and water were freely available throughout the study. All efforts were made to reduce animal suffering including minimising manipulations and defined endpoints limited to a moderate severity rating. All animals were humanely euthanised at the end of each experiment by inducing unconsciousness using isoflurane gaseous anaesthesia, followed by cervical dislocation.

In total, 132 mice were used across the full study. No formal a priori sample size calculations were performed to power each study. Group sizes were informed by previous CCHFV vaccine challenge studies using the IFNAR^−/−^ model and were selected in accordance with the principles of Replacement, Reduction and Refinement (3Rs). Batches of animals were given a minimum of one week to acclimatise prior to being allocated to treatment groups using individual animal identification numbers, with assignment undertaken randomly by animal facility staff. Investigators were not blinded to treatment allocation during vaccination, clinical assessment, or laboratory analyses; histopathological assessment was performed by a pathologist blinded to study group allocation. No animals were excluded from the study. All available data were included in the relevant analyses; where individual samples could not be included due to insufficient sample material, the reason and corresponding sample sizes are specified in the relevant figure legends.

### Cells and viruses

HEK293T/17 cells (ATCC, cat. no. CRL-11268; RRID: CVCL_1926) and Huh-7 cells (ATCC, cat. no. PTA-4583; RRID: CVCL_0336) were maintained at 37 °C with 5% CO_2_, and propagated in complete Dulbecco's Modified Eagle's Medium (DMEM-10), containing 10% foetal bovine serum (FBS), 1% L-glutamine, and 1% penicillin-streptomycin. All cell lines used in this study were confirmed negative for mycoplasma contamination. No additional cell-line identity authentication was performed following receipt from the indicated sources. CCHFV strain IbAr10200 (GenBank accession numbers NC_005302.1, MH483988.1, and AY947891.1 for the S, M, and L segments, respectively) was initially harvested from suckling mouse brain homogenates and subsequently passaged on SW13 cells (ECACC, cat. no. 87031801; RRID: CVCL_0542). Viral stocks were quantified by focus-forming assay in Vero E6 cells (ECACC, cat. no. 85020206; RRID: CVCL_0574) using a rabbit anti-CCHFV NP primary antibody (United States Biological, cat. no. 299578-USB; obtained via Stratech Scientific) followed by a goat anti-rabbit IgG peroxidase-conjugated secondary antibody (Sigma–Aldrich, cat. no. A0545). A dose-ranging study in A129 IFNAR^−/−^ mice established the lowest lethal dose (LD100) as 10 focus-forming units (FFU) administered in a 100 μL volume. Virus stocks were titrated prior to use to confirm infectious titre stability.

### Construction of ChAdOx2 vaccine candidates

For candidate generation, ChAdOx2 replication-defective E1/E3 deleted chimpanzee adenovirus vectors were produced from the wild-type replication-competent isolate AdC68, with further modification to the E4 region. For the NP-encoding candidate, the gene coding the open reading frame nucleic acid sequence of the CCHFV IbAr10200 S segment (GenBank accession number NC_005302.1) was codon-optimised for expression in humans and inserted into a shuttle plasmid between a truncated version of the human cytomegalovirus major immediate-early promoter (sCMV), which excludes intron A and two tetracycline operator 2 sites, and the bovine growth hormone polyadenylation signal. GPC-encoding vaccine candidates were designed to express the full GPC, Gc or Gn glycoprotein subunits, or secreted GP38. The nucleic acid sequence of the CCHFV IbAr10200 M Segment (GenBank accession number NC_005300.2) was codon-optimised for expression in humans and sequences encoding the full GPC, Gc, Gn, or GP38 synthesised (GeneArt, Thermo Fisher Scientific). Gn and Gc antigen sequences were designed to include the first 20 amino acids of the native GPC signal peptide followed by a unique 10-amino-acid linker recreating the natural Ski-1/S1P cleavage junctions. The Gn insert encoded the 350-amino-acid Gn region terminating upstream of the NSm region, while the Gc insert spanned the remaining 644 amino acids to the GPC stop codon. The GP38 insert encoded the 268-amino-acid secreted protein with an added N-terminal tissue plasminogen activator leader. Sequences encoding GPC, Gc, and Gn were cloned into shuttle plasmids under the control of the sCMV promoter, whereas the GP38 insert was cloned under the control of the full-length CMV immediate-early promoter (lCMV), followed by the bovine growth hormone polyadenylation signal. The antigen expression cassettes were then inserted into the E1 locus via site-specific recombination technology between the ChAdOx2 destination DNA BAC vector and the shuttle plasmid. The viral vectors were then rescued and grown in the HEK-293 derived T-Rex™-293 cell line (Invitrogen, cat. no. R71007; RRID: CVCL_D585) prior to caesium chloride (CsCl) purification and sterile filtration for the production and titration of the final vectors. The complete genome sequences of the generated vaccine candidates were confirmed by next-generation sequencing.

### SDS-PAGE and Western blot analysis

HEK293T/17 cells were seeded at 5 × 10^5^ cells per well in 2 mL DMEM-10 in 6-well plates and incubated for 24 h (h) at 37 °C with 5% CO_2_. Cells were transduced with ChAdOx2 viral vectors diluted in Opti-MEM at a multiplicity of infection (MOI) of 3 in a final volume of 2 mL per well. After 48 h, culture supernatants and cells were collected. Cells were washed with cold phosphate buffered saline (PBS) and lysed in Pierce RIPA buffer supplemented with Halt Protease Inhibitor Cocktail (Thermo Fisher Scientific). Lysates were incubated on ice for 15–30 min (min) and clarified by centrifugation at 15,000×*g* for 15 min at 4 °C. Supernatants were centrifuged at 5000×*g* for 10 min and supplemented with protease inhibitors. All samples were stored at −80 °C until analysis.

Protein concentration in cell lysates was determined using the Pierce BCA assay (Thermo Fisher Scientific). Lysates were diluted in RIPA to yield 10 μg per lane and mixed 1:1 with 2 × Laemmli buffer (Bio-Rad). Recombinant proteins (diluted to 0.1–1 μg in PBS) were used as positive controls. For Gn detection, samples were prepared under reducing conditions (50 mM DTT); samples for detection of NP, Gc, and GP38 were prepared under non-reducing conditions. Samples were heated at 95 °C for 5 min and separated by SDS-PAGE using Mini-PROTEAN precast gels (Bio-Rad) in Tris-Glycine buffer.

Proteins were transferred to 0.2 μm nitrocellulose membranes using the Trans-Blot Turbo system (Bio-Rad). Immunodetection was performed using the iBind Western system (Thermo Fisher Scientific). The following primary antibodies were used at 1:500: anti-NP (mAb 9D5), anti-PreGc (mAb 11E7), and anti-GP38 (mAb 13G8), all obtained from the Joel M. Dalrymple-Clarence J. Peters USAMRIID Antibody Collection through BEI Resources, NIAID, NIH. An in-house-generated mouse anti-Gn polyclonal antibody was used at 1:1000, with Gn-specificity confirmed by ELISA and Western blot against recombinant CCHFV Gn (Native Antigen, cat. no. REC31615). HRP-conjugated anti-mouse IgG (1:1000, Bio-Rad) was used as the secondary antibody. Blots were developed with Clarity Max ECL reagent (Bio-Rad), and chemiluminescent signals were captured using a ChemiDoc Touch Imaging System. A colourimetric image was acquired to visualise the protein ladder.

### 13G8 capture ELISA

Maxisorp 96-well Nunc plates (Thermo Fisher Scientific) were coated overnight at 4 °C with the GP38-specific monoclonal antibody 13G8 (2 μg/mL in PBS) at 50 μL per well. Plates were washed with PBS containing 0.05% Tween-20 (PBS/T) and blocked with 200 μL/well of Blocker™ Casein (Thermo Fisher Scientific) for 1 h at room temperature (RT; ∼20 °C). Supernatants diluted 1:10 in casein were added in duplicate at 50 μL/well in a three-fold dilution series. A three-fold serial dilution of recombinant GP38 protein (1 μg/mL to 0.002 μg/mL in casein, Native Antigen Company, cat. no. REC32029) was included on each plate as a standard curve and incubated for 2 h at RT. After washing, plates were incubated with an in-house human polyclonal serum pool (1:500 dilution), generated following immunisation with a CCHFV GPC-expressing vaccine and confirmed to react with recombinant CCHFV GP38 by ELISA, followed by alkaline phosphatase (AP)-conjugated goat anti-human IgG (γ-chain specific; Sigma A3187) diluted 1:1000 in casein, each for 1 h at RT. Plates were washed and developed with 100 μL/well p-nitrophenyl phosphate (pNPP) substrate. Optical density (OD) values for each well were measured at 405 nm using a Bio-Tek ELx800 Microplate Reader.

### Mice, vaccinations, blood sampling, and challenge with CCHFV

Male and female immunocompromised A129 interferon-α/β receptor knockout (IFNAR^−/−^) mice (129Sv/Ev background) aged 6–9 weeks were used for the GPC-focused Study I (42 female and 42 male). For the NP-focused Study II, 48 female IFNAR^−/−^ mice were used; female-only groups were selected to minimise husbandry issues associated with male aggression and because no significant sex-associated differences in the humoural immunological outcomes assessed by sex were identified in Study I. Animals were obtained from Marshall BioResources (UK) (female: cat. no. AM-TRAN-F-5; male: cat. no. AM-TRAN-M-5). The ChAdOx2 vaccines were formulated in endotoxin-free PBS and administered by intramuscular injection into the caudal aspect of the hind leg. For both studies, groups of IFNAR^−/−^ mice were split into two cohorts (*n =* 6 for immunogenicity and *n =* 6 for challenge per regimen) and vaccinated with PBS, ChAdOx2-GFP, or ChAdOx2 vaccines expressing CCHFV antigens at a standard dose of 1 × 10^8^ infectious units (IU). A low-dose ChAdOx2-GPC group (1 × 10^6^ IU) was also included in Study I. Mice received a total volume of 50 μL. Terminal bleeds and spleen harvesting for immunogenicity assessment were taken at 21 days post-immunisation.

For the challenge of IFNAR^−/−^ mice, a 100 μL volume of 10 ffu CCHFV was intradermally administered in the upper medial area of the back, 21 days after the final vaccine dose was given. For the following 21 days, challenged mice were weighed and body temperatures recorded daily via a pre-inserted identification/temperature chip (idENTICHIP with Bio-Thermal, MSD), and clinical signs were assessed at least twice daily. Clinical scores were assigned numerical values for analysis purposes as follows: 0, normal; 2, ruffled fur or photosensitivity; 3, lethargy, arched posture, wasp-waisted appearance, or eyes closed; 5, laboured or rapid breathing; and 10, immobile or paralysed. In accordance with institutional animal welfare guidelines, animals were euthanised upon reaching predefined humane endpoints, defined as ≥20% weight loss from baseline challenge weight sustained for more than 24 h, development of neurological signs, immobility or inactivity (even upon stimulation), paralysis, or a combination of elevated clinical scores indicative of severe disease.

### Post-mortem examination and histopathology

A post-mortem examination was performed for all IFNAR^−/−^ mice euthanised post–challenge, either when reaching humane endpoints or at the scheduled end of the study. Samples of liver and spleen taken from each animal were fixed by immersion in 10% neutral-buffered formalin (NBF) for at least 7 days before being trimmed and processed to paraffin wax (FFPE blocks). 4 μm thick sections were cut and stained with haematoxylin and eosin (H&E), and digitally scanned in a Hamamatsu S360 scanner (Hamamatsu, Tokyo, Japan). Scanned slides were evaluated through ndp.view2 software (Hamamatsu, Tokyo, Japan) by a qualified pathologist blinded to the treatment of each animal and group details; e-slides were randomised prior to examination to prevent bias. Histopathological lesions in liver and spleen were evaluated using a semi-quantitative scoring system (0–4). Each tissue comprised two predefined lesion categories. For liver, hepatocellular necrosis and inflammatory infiltration were assessed; for spleen, lymphocytolysis and macrophage/neutrophil infiltration were assessed. In Study I, lesions within each tissue were evaluated in combination and assigned a single composite score (0–4). In Study II, the two lesion categories were scored independently (0–4 each) and combined to generate the overall histopathology score presented for each tissue. No weighting was applied between lesion categories.

Consecutive FFPE sections were stained using the *in-situ* hybridisation RNAscope method to detect CCHFV RNA in tissue sections using a Leica Bond RXm automated staining platform (Leica Biosystems). Briefly, tissues were pre-treated with hydrogen peroxide for 10 min, target retrieval for 15 min (98–102 °C), and Protease III for 15 min (40 °C) (all Advanced Cell Diagnostics). A CCHFV-specific RNAscope probe targeting the GPC of the IbAr10200 strain (Advanced Cell Diagnostics, cat. no. 479798; LS V-CCHFV-m-env-01) was incubated on the sections for 2 h at 40 °C. Signal amplification was performed according to the RNAscope protocol (ACD 2.5 Red Rev B, adapted for use with the V-CCHFV-m-env-01 LS probe) using the RNAscope 2.5 LS Reagent Kit-Red (Advanced Cell Diagnostics) and the BOND Polymer Refine RED detection system for chromogenic visualisation, followed by haematoxylin counterstaining (Leica Biosystems). Digital images were captured using a Hamamatsu S360 slide scanner and analysed with ndp.view2 software. The presence of viral RNA was semi-quantitatively scored as 0 (none), 1 (minimal), 2 (mild), 3 (moderate), or 4 (abundant) RNAscope staining. For Study II, Nikon NIS-Elements AR software (Nikon, Tokyo, Japan) was used for digital image analysis to quantify the percentage area positive for viral RNA within tissue sections.

### RT-qPCR

Viral load quantification by Reverse Transcription quantitative PCR (RT-qPCR) was performed as previously described.^21^ Tissue samples were weighed, resuspended in 1.5 mL PBS and homogenised through a Corning Netwell plate (440 μm mesh) using the barrel of a 2 mL syringe, and then the homogenate collected. 200 μL of tissue homogenate or blood was transferred to 600 μL RLT buffer (Qiagen) plus β-mercaptoethanol and, after at least 10 min, 600 μL 70% isopropanol added to each sample. Samples were then transferred from the CL-4 laboratory to a CL-3 laboratory where contents were transferred to new tubes for RNA extraction outside of containment. Tissues were further homogenised using a QIAshredder (Qiagen) at 16,000×*g* for 2 min. RNA was then extracted using either the KingFisher Flex automated platform with the BioSprint 96 One-For-All Vet Kit (Indical) or manually using the RNeasy Mini Kit (Qiagen), according to the manufacturers’ instructions. RNA was eluted in 100 μL AVE buffer (Indical). Samples were analysed by qRT-PCR using the TaqMan Fast Virus 1-Step Master Mix (ThermoFisher Scientific) in fast-cycling mode, with primers and probes targeting the CCHFV S Segment.^40^ Viral loads were quantified using a ten-fold serial dilution of synthetic CCHFV S segment RNA (Integrated DNA Technologies). Results are expressed as RNA copies per gram of tissue or per mL of blood, with limits of quantification (LoQ) indicated in the figures for each dataset.

### IFN-γ Enzyme-linked immunospot (ELISpot) assay

Evaluation of CCHFV-specific T-cell responses following vaccination was performed via IFN-γ ELISpot assays as previously described using CCHFV strain IbAr10200 peptides spanning the M segment (JPT Peptide Technologies) and mouse IFN-γ ELISpot reagents (Mabtech).^21^ For NP-induced responses, ELISpot assays were similarly performed using peptides spanning the IbAr10200 S segment (JPT Peptide Technologies), consisting of 15-mer peptides overlapping by 10 residues. Splenocytes were seeded at 2.5 × 10^5^ cells per well onto PVDF microtitre plates pre-coated with anti-mouse IFN-γ capture antibody AN18 (Mabtech) and stimulated with peptide pools at a final concentration of 2.5 μg/mL per peptide, with 20 peptides per pool. Media-only wells were included as negative controls, and PMA/ionomycin (eBioscience cell stimulation cocktail, Fisher Scientific, cat. no. 00-4970-03) was used as a positive control. Background spot counts from unstimulated wells were subtracted from peptide-stimulated wells, and data expressed as spot-forming units (SFU) per 10^6^ cells. Responses are presented either for individual peptide pools or summed across the target protein.

### TNF-α and IL-2 FluoroSpot assay

Splenocytes were seeded onto pre-coated FluoroSpot plates and stimulated with M segment peptides using the same methodology as the IFN-γ ELISpot assay, including media-only and PMA/ionomycin controls. Plates were incubated for 18 h at 37 °C, 5% CO_2_ in a humidified incubator before TNF-α and IL-2 responses were assessed using a multiplex FluoroSpot assay (Mabtech, cat. no. FSP-314245-2) according to the manufacturer's instructions. Briefly, plates were washed five times with PBS, and detection antibodies diluted in 0.1% BSA-PBS were added and incubated at RT for 2 h. Plates were washed again, followed by incubation with fluorophore-conjugated anti-TNF-α and anti-IL-2 detection antibodies, incubated for 1 h in the dark, and washed. Fluorescence enhancer was applied for 5–15 min before plates were dried overnight in the dark. Plates were analysed using a Mabtech Iris reader, and background spot counts from unstimulated wells were subtracted from peptide-stimulated wells. Data are expressed as SFU per 10^6^ cells.

### Standardised enzyme-linked immunosorbent assay (ELISA) for total IgG

Anti-CCHFV IgG against Gc and Gn were measured by in-house indirect ELISAs as described previously.^21^ In addition, we developed an in-house recombinant NP and a GP38 ELISA using the same methodology. Briefly, Nunc MaxiSorp 96-well plates (Thermo Fisher Scientific) were coated overnight at 4 °C with recombinant NP (His-tag) or GP38 (Strep-His tag) protein at a final concentration of 5 μg/mL in PBS (both proteins obtained from Native Antigen Company, cat. no. REC31639 and REC32029, respectively). Plates were washed with PBS/T and wells then blocked with 1% Blocker™ Casein in PBS for 1 h at RT. Mouse sera were initially diluted 1:100 in casein and further diluted as necessary when OD values exceeded 2.0 to ensure measurements fell within the linear range of the standard curve. A serial dilution of the standard positive sera pool for each antigen was added to the plates to produce ten standard points that were assigned arbitrary units (AUs). Two internal controls of the positive standard serum pool and wells containing casein alone were included on each plate. Plates were incubated for 2 h at RT on a plate shaker set to 320 revolutions per minute (rpm) and then washed and alkaline phosphatase (AP)-conjugated goat anti-mouse IgG (Sigma–Aldrich, A1418), diluted 1:5000 in casein, was added to all wells for 1 h at RT with 320 rpm shaking. Plates were washed and developed by addition of pNPP substrate. OD values were measured at 405 nm using an ELx808 absorbance reader (BioTek) and fitted to a four-parameter logistic (4 PL) model using Gen5 software version 3.09 (BioTek), and serum AUs were calculated by interpolating OD_405_ values from the standard curve and multiplying by the corresponding dilution factor.

### Normalised IgG subclass and avidity ELISAs

Testing of IgG subclasses and IgG antibody avidity towards CCHFV GP38 and Gc were performed as per the total IgG ELISA with minor modifications, as previously described.^21^ For subclass analysis, mouse sera were diluted in casein to 1 total IgG AU and incubated for 2 h at 37 °C. AP-conjugated goat anti-mouse IgG1, IgG2a, and IgG2b (Southern Biotech, cat. nos. 1071-04, 1081-04, and 1091-04) were used at a 1:4000 dilution, and AP-conjugated goat anti-mouse IgG3 (Abcam, cat. no. 98705) at 1:500 dilution. Plates were incubated for 1 h at 37 °C prior to development, and results are presented as OD_405_ values.

For IgG avidity assessment, sera normalised to 1 total IgG AU were treated with increasing concentrations of sodium thiocyanate (NaSCN) in 0.5 M increments (0–3 M) for 10 min at room temperature prior to secondary antibody incubation and development as described above. OD_405_ was measured until the internal positive control wells reached an OD_405_ of 1.0. Gen5 ELISA software v3.09 (BioTek) was used to calculate avidity using the intercept of the OD_405_ curve for each sample with the line of 50% reduction of the OD_405_ in the 0 M NaSCN wells and expressed as the half maximal inhibitory concentration (IC50).

### Pseudotype neutralisation assay using CCHFV TecVLP

Antibody neutralisation capacity was evaluated with the tecVLP neutralisation assay using IbAr10200,^41^ as previously described with minor modifications.^21^ Briefly, HEK293T/17 cells were transfected with CCHFV helper plasmids encoding the bacteriophage T7 RNA polymerase, strain IbAr10200 GPC, NP, the RNA-dependent RNA polymerase helper plasmids, and the pL-Luc minigenome plasmids (shared by Professor Aeric Bergeron, CDC, USA) and using TransIT-LT1 Transfection Reagent (Mirus Bio, cat. no. MIR2300). Antibody neutralisation capacity was evaluated in Huh-7 target cells seeded at 1 × 10^5^ cells/mL (1 × 10^4^/well) in white opaque 96-well microplates (Thermo Fisher Scientific) and incubated for 24 h at 37 °C. Heat-inactivated sera were diluted in two-fold dilutions in DMEM supplemented with 5% FBS (DMEM-5) within 96-well culture plates, with an initial sera dilution of 1:40 up to 1:2560, and then mixed at a 1:1 ratio with thawed tecVLP, diluted in DMEM-5 to give ∼5 × 10^5^ relative luminescence units (RLU), and incubated for 1 h at 37 °C with 5% CO_2_. The serum-tecVLP mixes were then transferred to the Huh-7 cells seeded the previous day, and incubated for 48 h at 37 °C. After 48 h, all media was removed from wells and RLU was measured by the addition of 30 μL per well of the Nano-Glo Luciferase Assay System (Promega), with luminescence intensity measured using a CLARIOstar Plus Microplate Reader (BMG Labtech). RLU data points for the dilution of sera were plotted using a 4-PL curve generated with GraphPad Prism software (version 10). Data were normalised and non-linear regression analysis used to determine the IC50 using GraphPad Prism.

### Fcγ receptor-binding Luminex bead-based assay

An in-house Luminex assay was used to quantify antigen-specific Fcγ receptor (FcγR)-binding. GP38 or Gc proteins (10 μg per 1.25 × 10^6^ beads, Native Antigen), as well as bovine serum albumin (BSA) as a negative control, were coupled to Bio-Plex magnetic COOH beads (Bio-Rad) using a two-step carbodiimide reaction according to the manufacturer's instructions (Bio-Plex Amine Coupling Kit). Coupled beads were added to 96-well plates (Greiner Bio-One) at 1 × 10^6^ beads/mL (50 μL/well) in 0.1% BSA/PBS-Tween and incubated for 30 min at RT on a plate shaker (700 rpm). Plates were washed twice with 0.1% BSA/PBS-Tween before addition of serum samples. Sera were diluted in casein to a concentration equivalent to 1 total IgG AU from the corresponding antigen-specific ELISA and analysed in duplicate. Plates were incubated for 1 h at 37 °C with shaking (700 rpm) and washed twice. FcγR binding was detected by addition of biotinylated mouse FcγRIII/CD16, FcγRIIB/CD32B, or FcγRIV/FCGR4 proteins (Sino Biological) at 0.5 μg/mL, followed by streptavidin-phycoerythrin (1:4000, Thermo Fisher Scientific) in 50 μL/well of 0.1% BSA/PBS-Tween. Following a 1 h incubation at RT with shaking, plates were washed three times, and 100 μL/well of MAGPIX Drive Fluid PLUS was added. Relative antigen-specific FcγR activity was acquired on a MAGPIX Multiplex Reader (Bio-Rad).

### Statistical analysis

All data were analysed using GraphPad Prism version 10 (GraphPad Software Inc., California, USA). Survival data were analysed using Kaplan–Meier curves, with differences assessed by log-rank (Mantel–Cox) tests comparing vaccinated groups to PBS-immunised controls. These animals served as the predefined negative control for efficacy comparisons, with ChAdOx2-GFP included as an additional vector control. No formal comparisons of survival or other challenge outcomes between sexes were performed because the number of animals of each sex within individual challenge groups was insufficient for meaningful statistical comparison. Data normality was assessed using the Shapiro–Wilk test. Immunological measurements were analysed using Kruskal–Wallis tests with Dunn's correction for multiple comparisons when assessing differences across multiple vaccine groups, or Mann–Whitney tests with Holm-Šídák correction for multiple comparisons between sexes or two vaccine-groups. Principal component analysis (PCA) was performed as an exploratory multivariate analysis in Python (version 3.12.7, Anaconda distribution) using the scikit-learn package (StandardScaler and PCA functions) and visualised using the matplotlib library.

Statistical tests used for each dataset are indicated in the corresponding figure legends. For multi-group comparisons, only statistically significant and biologically relevant comparisons are annotated in the figures; complete statistical outputs for survival and immunological analyses are provided in Supplementary Tables S1–S7. *p* values < 0.05 were considered statistically significant.

### Role of funders

The funder had no role in the study design, data collection, data analysis, interpretation of the data, manuscript preparation, or the decision to publish.

## Results

### Design and robust expression of individual CCHFV antigens (GP38, Gn, Gc and NP) by ChAdOx2 vaccine candidates

The GPC-driven protective mechanisms for CCHFV have not yet been fully established. Given the protection previously demonstrated for ChAdOx2-GPC, we generated three new ChAdOx2 constructs to elucidate the protective nature and resulting immunogenic drivers of the individual GPC domains; GP38, Gn, and Gc from strain IbAr10200 (Fig. 1A). The first two candidates, ChAdOx2-Gn and ChAdOx2-Gc, encoded the Gn or Gc structural glycoproteins with each retaining the native GPC signal peptide. The third novel construct, ChAdOx2-GP38, was designed for expression of the ∼38 kDa secreted GP38 protein in the absence of the upstream mucin-like domain (MLD), using an N-terminal tissue plasminogen activator (tPA) leader in place of the native signal peptide used in the Gn and Gc constructs.

**Fig. 1:**
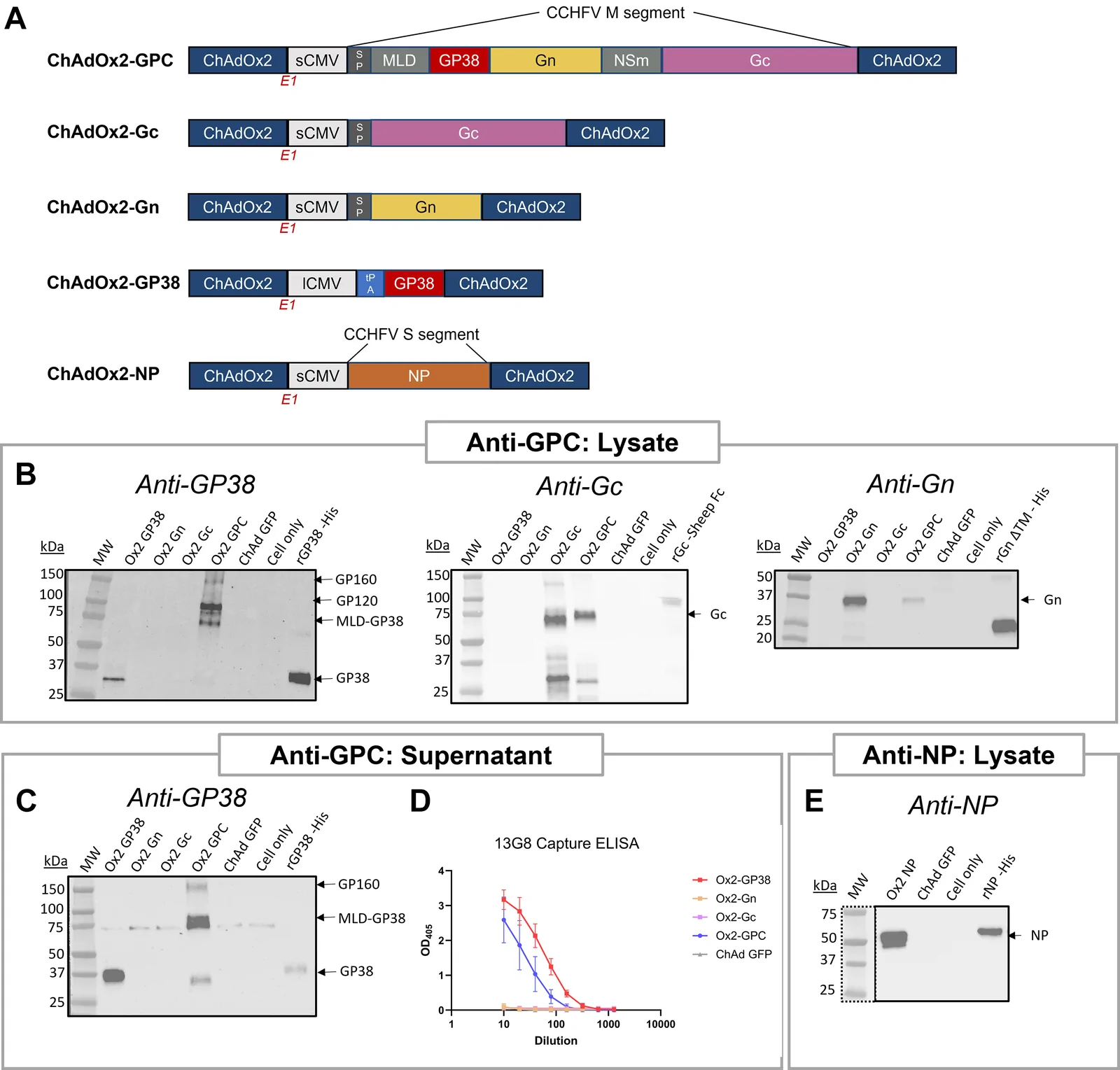
**Design of ChAdOx2 vectors capable of expressing CCHFV GPC subunits or NP. (A)** Schematic representation of ChAdOx2 candidates designed to express either the full-length CCHFV glycoprotein precursor (GPC), individual GPC subunits (GP38, Gn, or Gc), or the S-segment encoded nucleoprotein (NP). Antigenic inserts are encoded at the E1 locus of the replication-deficient ChAdOx2 genome and are expressed under the control of either a shortened (sCMV) or full-length (lCMV) human cytomegalovirus immediate-early promoter, with all constructs using sCMV except GP38, which uses lCMV. The GP38 construct incorporates an N-terminal tissue plasminogen activator (tPA) leader, replacing the native signal peptide (SP) retained in the Gn, Gc, and GPC constructs; NP lacks a signal peptide and is expressed intracellularly. Western blots of GPC-based vector transduced cell lysates **(B)** and clarified supernatants **(C)** that were resolved by SDS-PAGE, transferred to nitrocellulose and probed by anti-GP38 13G8 monoclonal antibody (mAb), mouse polyclonal anti-Gn antibodies, and anti-Gc 11E7 mAb. Recombinant GP38 and Gc with His- or sheep-Fc purification tags, respectively, were included as positive controls; the recombinant Gn (rGn ΔTM) corresponds to the soluble ectodomain with the transmembrane region deleted resulting in an observed molecular weight of ∼25 kDa. **(D)** GP38 capture ELISA on clarified culture supernatants assayed using plates coated with mAb 13G8; optical density was read at 405 nm. Data represent the mean ± SD of four replicates across two independent experiments. **(E)** Western blot of ChAdOx2-NP transduced cell lysates probed with anti-NP 9D5 mAb. Recombinant NP protein was included as a positive control. Dotted lines indicate where the blot was cropped to remove non-essential regions. Molecular-mass markers (kDa) are shown at left of each panel.

*In vitro* expression of encoded CCHFV antigens from each vector was confirmed by immunoblotting from transduced HEK-293T cells (Fig. 1B and C). An anti-GP38 mAb detected the expected ∼38 kDa band in ChAdOx2-GP38 lysate, whereas the ChAdOx2 full-length GPC lysate predominantly contained higher-molecular-weight GP38-containing species consistent with MLD-GP38 processing intermediates (∼85 kDa and above), with the ∼38 kDa band markedly reduced (Fig. 1B). Probing with polyclonal anti-Gn antibodies showed a dominant ∼37 kDa band confined to the ChAdOx2-Gn and ChAdOx2-GPC lysate, with stronger intensity in the Gn-only lysate (Fig. 1B). Detection with an anti-Gc mAb confirmed robust expression of the mature ∼75 kDa Gc in both ChAdOx2-Gc and ChAdOx2-GPC lysates, with minor size variation and additional lower-molecular-weight bands likely representing partial cleavage products (Fig. 1B). Analysis of supernatants demonstrated secretion of GP38 from both ChAdOx2-GP38 and full-length GPC constructs, with stronger enrichment of the ∼38 kDa GP38 band in ChAdOx2-GP38-transduced cells and predominance of MLD-GP38 species in ChAdOx2-GPC cells (Fig. 1C). Capture ELISA similarly showed detectable GP38 in supernatants from both the GP38-only construct and ChAdOx2-GPC, with levels relatively lower in the full-length GPC construct (Fig. 1D).

To evaluate the nucleoprotein (NP) as a protective antigen in this adenoviral platform, we also generated a ChAdOx2 construct expressing the full-length open reading frame of the CCHFV S-segment encoding NP from the IbAr10200 strain (Fig. 1A). As NP is an intracellular protein, no signal peptide sequence was included. Detection with an anti-NP mAb confirmed robust expression of the ∼50–55 kDa NP antigen in lysate of ChAdOx2-NP-transduced HEK-293T cells (Fig. 1E). Following the demonstration of antigen expression from each of the subunit vaccines, immunogenicity and challenge studies in animals were initiated.

### Single-dose ChAdOx2 vaccines expressing GP38, Gc, or NP protect mice against lethal CCHFV challenge, with minimal pathology and reduced viral RNA levels

To evaluate the protective efficacy of the engineered GPC-based ChAdOx2 CCHFV vaccines, we conducted a challenge study in the immunocompromised interferon-α/β receptor 1 knockout (IFNAR^−/−^) A129 mouse model (*n* = 6 per group). The single-dose immunisation and challenge schedule for Study I is shown in Fig. 2A. This study also included a low-dose ChAdOx2-GPC group at 1 × 10^6^ infectious units (IU), alongside the standard 1 × 10^8^ IU GPC dose. Based on prior murine dose-ranging studies of adenoviral-vectored vaccines, this reduced dose was expected to induce a lower level of immunogenicity across the GPC and was therefore used to identify immune responses critical for protection in small animal models.^42^, ^43^, ^44^ Twenty-one days after immunisation, mice were challenged with a lethal dose of CCHFV strain IbAr10200.

**Fig. 2:**
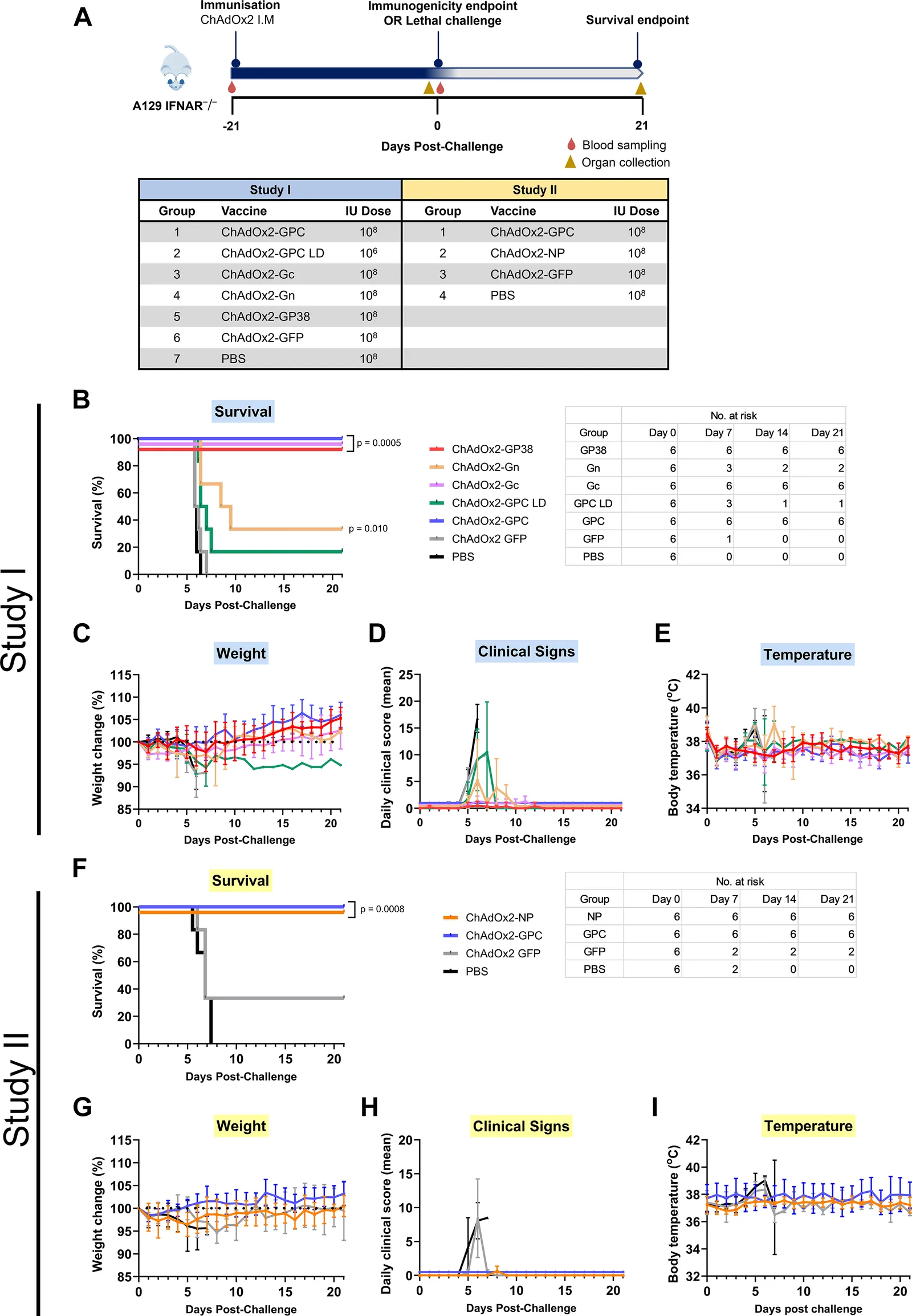
**ChAdOx2 vaccines expressing GPC-based or NP antigens protect IFNAR^−/−^ mice against lethal CCHFV challenge. (A)** Study schematic and design. A129 IFNAR^−/−^ mice (*n* = 12 per group) were immunised with ChAdOx2 vectors expressing GP38, Gn, Gc, or full-length GPC at a standard dose of 1 × 10^8^ infectious units (IU), a lower-dose full-length GPC formulation (ChAdOx2-GPC LD; 1 × 10^6^ IU), or negative controls consisting of vector-only (GFP) or PBS. Three weeks post-immunisation, mice were divided into two cohorts (*n =* 6 per cohort). One cohort was euthanised prior to challenge, and spleens were collected for assessment of cellular immune responses. The second cohort was challenged intradermally with CCHFV strain IbAr10200 and monitored daily for 21 days thereafter. Blood samples were collected at defined time points to assess humoural immune responses, and liver and spleen were collected at humane endpoint or day 21 post–challenge for viral load and pathogenesis analyses. A second study evaluated the ChAdOx2-NP construct alongside ChAdOx2-GPC and negative control groups under the same experimental conditions. **(B** and **F)** Survival curves following CCHFV challenge, with numbers at risk shown at days 0, 7, 14, and 21 post–challenge. **(C** and **G)** Percentage body weight changes relative to day 0. **(D** and **H)** Clinical scores shown as the daily mean of twice-daily assessments using the numerical scoring system described in Methods. **(E** and **I)** Body temperature changes (°C). Data are shown as mean ± SD per group for longitudinal measures at each timepoint. Survival significance was assessed using the log-rank (Mantel–Cox) test, with comparisons against the PBS control group. Exact p-values are displayed for statistically significant comparisons shown in panels **B** and **F**; complete log-rank test results and daily numbers at risk are provided in Supplementary Table S1.

Complete protection against lethal disease was achieved in animals receiving the ChAdOx2-GP38 or ChAdOx2-Gc subunit vaccines, as well as in those given the standard-dose ChAdOx2-GPC vaccine, with all animals surviving challenge (100%, 6/6 per group; Fig. 2B). In contrast, PBS- and ChAdOx2-GFP-immunised control mice rapidly developed severe disease and reached humane endpoints by days 6–8. Partial survival was observed in the ChAdOx2-Gn group, with 33.3% survival (2/6), which was significantly improved compared with PBS-immunised controls (*p* = 0.010), whereas survival in the low-dose ChAdOx2-GPC group was limited to 16.7% (1/6) and did not differ significantly from PBS controls (*p* = 0.062). Clinical observations were consistent with survival outcomes: all control animals developed overt disease, as did the majority of animals in the Gn (4/6) and low-dose GPC (5/6) groups, which exhibited marked weight loss, high clinical scores, and temperature fluctuations from days 5–7 post–challenge (Fig. 2C–E; Supplementary Figure S1). In contrast, ChAdOx2-GP38, ChAdOx2-Gc, and standard-dose GPC recipients maintained or increased body weight, had stable body temperatures, and displayed minimal clinical signs throughout the 21-day period, with only rare and transient weight loss or clinical signs observed (≤1/6 animals per group).

To evaluate the protective capacity of NP, a second challenge experiment was conducted (Study II) in which mice received a single dose of ChAdOx2-NP or ChAdOx2-GPC, and were challenged 21 days later under the same conditions (*n =* 6 per group). PBS- and ChAdOx2-GFP-immunised control mice similarly developed clinical signs from day 5 post–challenge, with all PBS mice succumbing (0/6 survival) and 4/6 ChAdOx2-GFP mice reaching humane endpoints between days 6–8; two GFP-immunised animals remained asymptomatic and survived to study endpoint (Fig. 2F–I). While survival did not differ significantly between PBS- and ChAdOx2-GFP-immunised control groups (*p* = 0.286), all mice immunised with ChAdOx2-NP or ChAdOx2-GPC survived infection (6/6 in each group), demonstrating significantly improved survival compared with PBS-immunised controls (*p* = 0.0008 for both comparisons). Clinical scores and physiological parameters mirrored these outcomes: ChAdOx2-NP and ChAdOx2-GPC recipients showed minimal clinical signs, stable body temperatures, and gradual weight gain, with only brief clinical signs or weight loss in a single NP-immunised animal that resolved by day 9 (Fig. 2G–I; Supplementary Figure S2).

We next assessed the extent of tissue histopathology and viral dissemination in vaccinated animals by examining formalin-fixed sections of liver and spleen collected either upon reaching humane endpoint or at the scheduled study endpoint 21 days post–challenge. In Study I, all PBS- and ChAdOx2-GFP-immunised mice, as well as animals in the ChAdOx2-Gn and low-dose (1 × 10^6^ IU) ChAdOx2-GPC groups that met humane endpoints during days 5–9 post–challenge, exhibited severe hepatic and splenic lesions (Fig. 3A). These lesions were characterised by multifocal hepatocyte necrosis and inflammatory cell infiltration in the liver, and lymphocyte depletion with infiltration of macrophages and polymorphonuclear leukocytes within the splenic red pulp (Supplementary Figure S3A–E). In contrast, surviving animals, including those in these partially protective groups and all mice vaccinated with ChAdOx2-GP38, ChAdOx2-Gc, or standard-dose ChAdOx2-GPC, displayed minimal to no histological abnormalities at 21 days post–challenge (Fig. 3A; Supplementary Figure S3F–H).

**Fig. 3:**
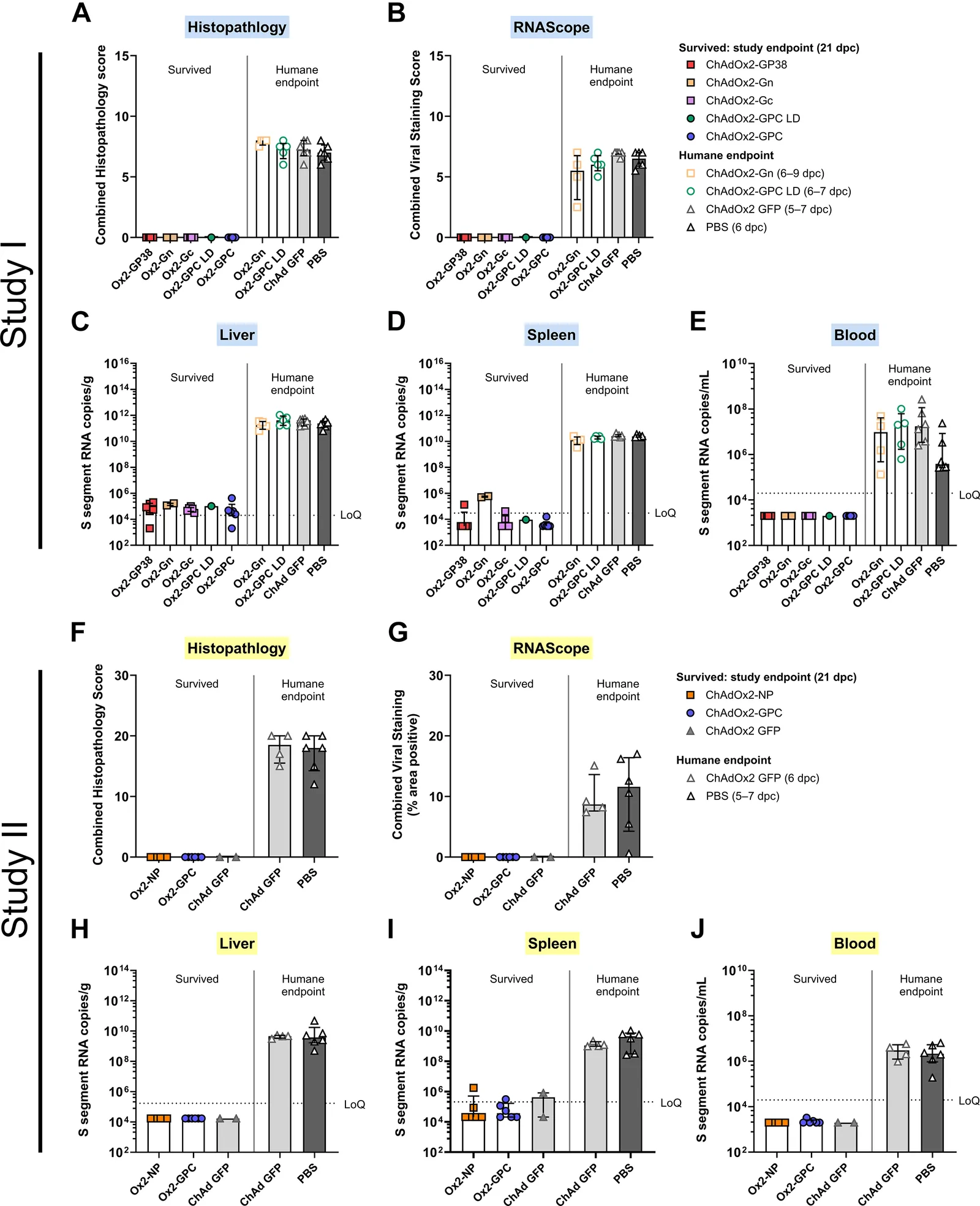
**ChAdOx2 vaccines expressing GP38, Gc, full-length GPC, or NP reduce histopathology and CCHFV RNA levels in IFNAR^−/−^ mice.** Tissues were collected from animals that survived to the scheduled study endpoint (21 days post–challenge [dpc]) or upon reaching humane endpoint for pathological and virological analysis. Study I: **(A)** Combined histopathology scores in liver and spleen; **(B)** combined viral RNA staining scores assessed by RNAscope in situ hybridisation; **(C**–**E)** viral RNA levels in liver, spleen, and blood quantified by RT-qPCR. Mice were vaccinated with ChAdOx2-GP38, -Gn, -Gc, -GPC (standard or low dose), ChAdOx2-GFP, or PBS (*n* = 6 per group) and challenged with CCHFV strain IbAr10200. Study II: **(F)** Combined histopathology scores in liver and spleen; **(G)** viral RNA staining scores assessed by RNAscope; **(H**–**J)** viral RNA levels in liver, spleen, and blood quantified by RT-qPCR. Mice were vaccinated with ChAdOx2-NP, ChAdOx2-GPC, ChAdOx2-GFP, or PBS (*n* = 6 per group) and challenged under identical conditions. Histopathology scores were assigned in a blinded manner using the semi-quantitative scoring systems described in Methods, with combined tissue scores presented as described. RNAscope staining represents semi-quantitative assessment of viral RNA signal. RT-qPCR data are presented as S-segment RNA copies per g (tissue) or mL (blood), with the dotted line indicating the limit of quantification (LoQ). Scatter plots show individual animals with group medians and interquartile ranges. Across all panels, the grey vertical line separates surviving animals sampled at the scheduled 21-dpc study endpoint from those sampled upon reaching humane endpoint; filled symbols represent study-endpoint animals and open symbols represent humane-endpoint animals. Group-specific humane-endpoint sampling ranges are indicated in the figure keys.

CCHFV RNA detection by RNAscope parallelled histopathology findings. Abundant viral RNA was detected in liver and spleen from control-immunised animals and from ChAdOx2-Gn and low-dose GPC mice that met humane endpoints (Fig. 3B; Supplementary Figure S3A–E). In contrast, no detectable viral RNA was observed at 21 days post–challenge in tissues from mice immunised with ChAdOx2-GP38, ChAdOx2-Gc, or standard-dose ChAdOx2-GPC (Fig. 3B; Supplementary Figure S3F–H). Quantitative RT-qPCR analysis of liver, spleen, and blood showed a similar pattern, with viral RNA levels at or below the limit of quantification (LoQ) in surviving animals sampled at 21 days post–challenge, indicating minimal detectable viral RNA, whereas high viral RNA levels were detected in Gn- and low-dose GPC-immunised mice that reached humane endpoints, as well as in GFP- and PBS-immunised controls (Fig. 3C–E).

The subsequent study assessing ChAdOx2-NP immunisation showed a similar pattern, with PBS- and ChAdOx2-GFP immunised mice that met humane endpoints during days 5–7 post–challenge exhibiting severe hepatic and splenic lesions accompanied by high levels of viral RNA staining, except for the two ChAdOx2-GFP animals that survived to 21 days post–challenge without marked pathology (Fig. 3F and G; Supplementary Figure S4A–C). In contrast, mice vaccinated with either ChAdOx2-NP or ChAdOx2-GPC had minimal histopathology and no detectable viral RNA by RNAscope at 21 days post–challenge (Fig. 3F and G; Supplementary Figure S4A, D and E). RT-qPCR analysis of liver, spleen, and blood demonstrated viral RNA levels at or below the LoQ in ChAdOx2-NP and ChAdOx2-GPC-immunised animals sampled at 21 days post–challenge, whereas high viral RNA levels were detected in control animals that reached humane endpoints (Fig. 3H–J). Taken together, these results demonstrate that a single dose of ChAdOx2 vectors expressing GP38, Gc, or NP resulted in 100% survival following lethal challenge, comparable to the full-length GPC vaccine, whereas ChAdOx2-Gn alone or a 1 × 10^6^ IU dose of ChAdOx2-GPC afforded limited efficacy. Animals in these effective vaccine groups survived to the 21-day study endpoint, at which time viral RNA levels were at or below the LoQ, prompting further investigation into deconvoluting the immune mechanisms underlying ChAdOx2-mediated protection against CCHFV.

### ChAdOx2-GP38 and ChAdOx2-Gc vaccinations elicit robust antigen-specific IgG responses that are impaired in the lower-dose ChAdOx2-GPC group

To identify the immune drivers of the GPC-mediated protection outcomes described above, immunogenicity assessments were undertaken during the same vaccination-challenge experiment. As summarised in Fig. 2A, immunised IFNAR^−/−^ mice (*n* = 12/group) were divided into two subsets: animals culled on day 21 post-immunisation for cellular assays (*n* = 6), and the challenge subset (*n* = 6). This design enabled direct comparison of pre-challenge humoural immunity with subsequent survival outcomes, alongside a parallel cohort for assessments of *ex vivo* T-cell immunity.

We first examined antibody responses to the GPC-based ChAdOx2 candidates to determine which features distinguished protection against lethal CCHFV challenge from non-protected groups. Sera collected from mice prior to challenge were examined for binding and functional antibody activity. GP38-specific IgG levels were highest in the ChAdOx2-GP38 subunit group and were also readily detected in all standard-dose GPC-immunised mice; the two groups did not differ significantly from each other but both significantly exceeded GFP controls (GP38 vs GFP, *p* = 0.0006; GPC vs GFP, *p* = 0.0457; Fig. 4A). Notably, GP38-specific IgG responses were largely absent in the low-dose GPC group, with only one mouse exhibiting detectable IgG towards GP38, which corresponded to the sole survivor after challenge (Fig. 4A). Consistent with our previous work (Saunders et al., 2023), both dose groups of the ChAdOx2-GPC vaccine elicited little to no detectable anti-Gn IgG, and no response was detected following ChAdOx2-Gn subunit vaccination (Fig. 4B). The ChAdOx2-Gc vaccine induced the highest Gc-specific IgG titres, followed closely by the standard-dose ChAdOx2-GPC construct; both groups were significantly higher than GFP controls, with no significant difference between them (Gc vs GFP, *p* = 0.0008; GPC vs GFP, *p* = 0.0399; Fig. 4C). In line with the dose-dependent effect observed for GP38, the low-dose GPC group mice showed no detectable Gc-specific IgG relative to the standard dose (Fig. 4C). No significant sex-associated differences in anti-GP38 or Gc-specific IgG titres were observed in either the immunogenicity or challenge cohorts (all adjusted *p* > 0.05; Supplementary Figure S5A–C).

**Fig. 4:**
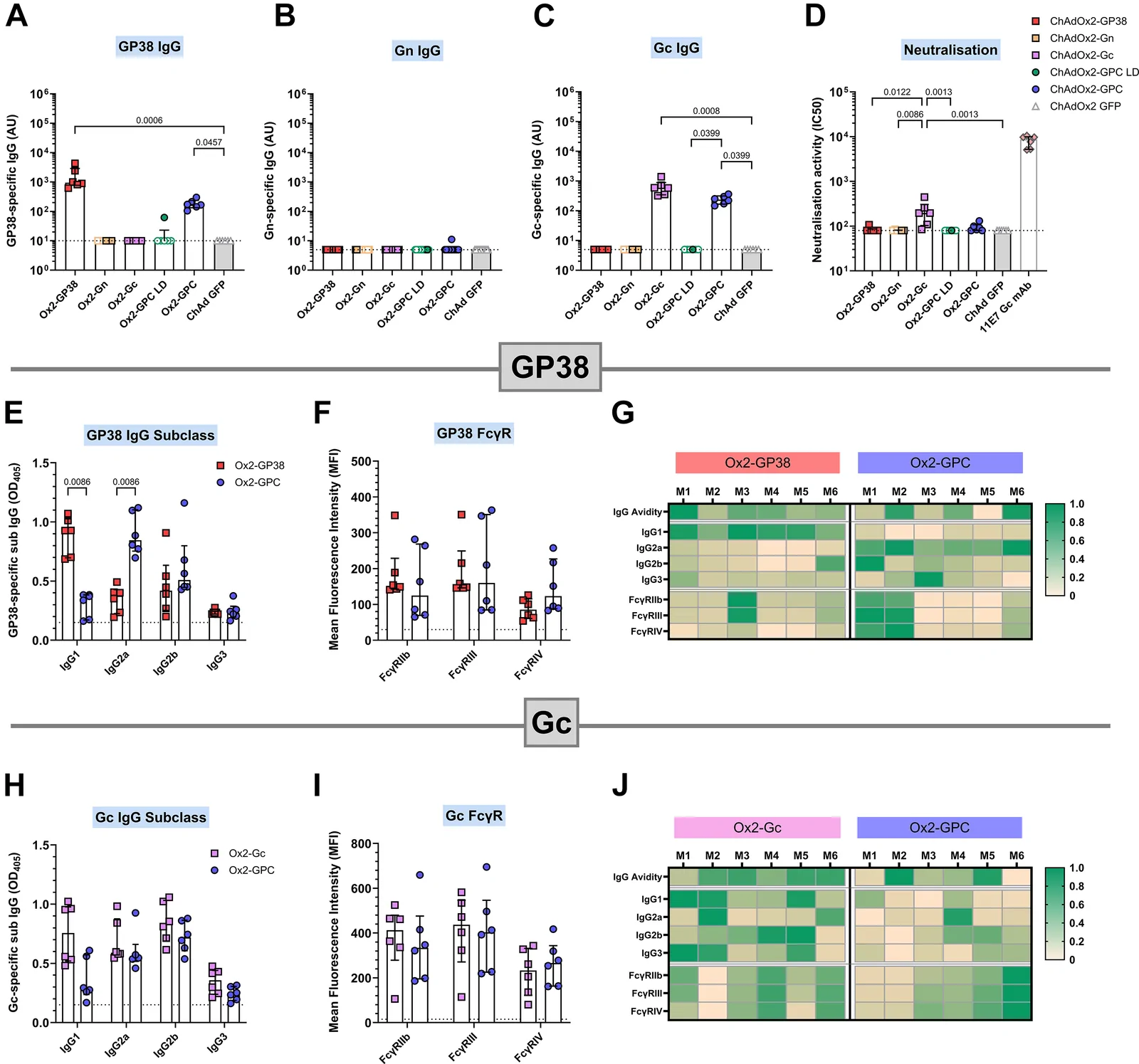
**Protective ChAdOx2-Gc and ChAdOx2-GP38 vaccines elicit strong antigen-specific IgG responses comparable to standard-dose ChAdOx2-GPC, with minimal neutralising activity.** IFNAR^−/−^ mice were vaccinated with a single dose of ChAdOx2 vaccines encoding CCHFV GPC antigens, or with ChAdOx2-GFP (*n =* 6 per group), and challenged three weeks later. Day-21 post-immunisation sera from mice subsequently challenged with CCHFV were analysed for CCHFV-specific humoural responses and assayed for **(A)** GP38-specific IgG, **(B)** Gn-specific IgG, and **(C)** Gc-specific IgG. Antibody titres were quantified by standardised ELISAs and data are expressed as arbitrary units (AU). **(D)** Neutralising antibody titres were determined using a pseudovirus neutralisation assay with CCHFV tec-VLPs and are expressed as IC_50_ values; the Gc-specific monoclonal antibody 11E7 served as a positive control. Antibody functionality in protective groups was further assessed. **(E**–**G)** GP38-specific IgG subclass distribution (E), Fcγ receptor-binding profiles (F), and a normalised heatmap of measured antibody features (G), comparing ChAdOx2-GP38 with standard-dose ChAdOx2-GPC. **(H**–**J)** Gc-specific IgG subclass distribution **(H)**, Fcγ receptor-binding profiles **(I)**, and a normalised heatmap of measured antibody features **(J)**, comparing ChAdOx2-Gc with standard-dose ChAdOx2-GPC. IgG subclasses were determined by ELISA using subclass-specific secondary antibodies, and Fcγ receptor binding was measured using fluorescently coded microspheres. Individual data points are shown as scatter plots with bars indicating the median and interquartile range. Dotted lines indicate the assay limit of quantification. Statistical significance was assessed using Kruskal–Wallis tests with Dunn's multiple-comparisons test for multi-group analyses **(A**–**D)** and Mann–Whitney tests with Holm-Šídák correction for multiple comparisons for two-group analyses **(E** and **F**, **H** and **I)**. Exact p-values are shown for statistically significant comparisons. In panels **A**–**C**, statistical annotations are shown only for selected biologically relevant comparisons that reached statistical significance; complete statistical results for all comparisons are provided in the Supplementary Table S2.

Neutralising activity, measured using the CCHFV tecVLP assay, was absent or low in the majority of groups (Fig. 4D). Quantifiable neutralising IC_50_ values were only detected in ChAdOx2-Gc immunised mice, which showed significantly higher neutralising titres than all other groups (all adjusted *p* < 0.05; Fig. 4D). Cumulatively, these data demonstrate that robust but largely non-neutralising GP38 and Gc IgG responses were elicited across protected mice, whereas the 1 × 10^6^ IU ChAdOx2-GPC and ChAdOx2-Gn groups exhibited minimal antibody titres and substantially lower survival.

### GP38- and Gc-specific antibodies from ChAdOx2 vaccination exhibit similar Fcγ receptor binding potential despite IgG subclass differences

We hypothesised that the reduced antibody levels induced by the 1 × 10^6^ IU ChAdOx2-GPC vaccination contributed to the loss of protection, whereas the elicited antibodies in the GP38-, Gc-, and standard-dose GPC protected groups mediated protective immunity. Thus, we next characterised the antibody profiles in these protected vaccine groups to identify humoural features associated with protection. Specifically, we assessed the GP38- or Gc-specific antibody profiles in their respective subunit vaccine groups and compared them with the corresponding responses induced by the full-length ChAdOx2-GPC vaccine at 1 × 10^8^ IU.

Comparative profiling of GP38-induced humoural responses initially through IgG subclass analysis demonstrated that the ChAdOx2-GP38 group exhibited significantly higher IgG1 levels, whereas the ChAdOx2-GPC group showed significantly higher IgG2a responses (both adjusted *p* = 0.0086; Fig. 4E). No clear differences were observed in IgG2b or IgG3 levels. ChAdOx2-GP38 mice also exhibited significantly lower IgG2a/IgG1 and IgG2b/IgG1 subclass ratios compared with ChAdOx2-GPC mice (Supplementary Figure S5D). This divergence in IgG subclass usage suggests that antigen format, specifically whether GP38 is secreted alone or presented in the context of the full GPC polyprotein, may influence T helper cell polarisation, with GP38 eliciting a Th2-like profile (IgG1) and GPC favouring a Th1-like bias (IgG2a). GP38-specific IgG avidity was comparable between the two groups (Supplementary Figure S5E). Binding of antigen-specific IgG to Fcγ receptors is indicative of the potential for antibody-mediated Fc effector functions; in mice, FcγRIII and FcγRIV are activating receptors associated with functions such as antibody-dependent cellular cytotoxicity and phagocytosis, whereas FcγRIIb is the sole inhibitory Fcγ receptor that regulates these responses.^45^, ^46^ Testing sera for binding to the Fcγ receptors FcγRIIb, FcγRIII, and FcγRIV showed no significant group-level differences for GP38, although some inter-animal variation was observed (all adjusted *p* > 0.05; Fig. 4F). Integration of antibody features in the normalised heatmap highlighted distinct response patterns between ChAdOx2-GP38 and ChAdOx2-GPC groups, with complementary principal component analysis (PCA) showing ChAdOx2-GP38 animals clustering with IgG1-associated features and ChAdOx2-GPC animals aligning more closely with IgG2a and FcγRIV (Fig. 4G; Supplementary Figure S5F). FcγRIIb and FcγRIII responses were intermediate across groups, suggesting these interactions are shared and not major contributors to the observed differences.

Characterisation of Gc-induced humoural immunity in protected ChAdOx2-Gc and ChAdOx2-GPC mice revealed Gc-specific IgG subclass levels were largely similar between groups, with both eliciting broadly distributed subclasses skewed toward a Th1-associated IgG2a/IgG2b bias (Fig. 4H). ChAdOx2-Gc mice displayed a modest trend toward higher Gc-specific IgG1 levels than in the ChAdOx2-GPC group, though not significant (*p* > 0.05), and showed modestly lower, but not significantly different (*p* > 0.05), IgG2a/IgG1 and IgG2b/IgG1 ratios compared with ChAdOx2-GPC mice, suggesting a trend towards a less strongly Th1-skewed response (Supplementary Figure S5G). Moreover, ChAdOx2-Gc and ChAdOx2-GPC sera exhibited comparable avidity of IgG against Gc (Supplementary Figure S5H). Likewise, Gc-specific sera from both groups showed similar binding to FcγRIIb, FcγRIII, and FcγRIV (Fig. 4I). The corresponding heatmap demonstrated substantial similarity in antibody feature profiles (Fig. 4J), and PCA likewise showed overlapping clustering between ChAdOx2-Gc and ChAdOx2-GPC mice, indicating broadly similar humoural responses (Supplementary Figure S5I).

Together, these findings indicate that the non-neutralising GP38-specific antibodies elicited by ChAdOx2-GP38 are predominantly IgG1-skewed. In contrast, ChAdOx2-GPC vaccination generated Th1-associated IgG2a-dominated responses to both GP38 and Gc, while ChAdOx2-Gc induced a broadly similar but less strongly Th1-skewed IgG subclass profile against Gc. Across all vaccine groups, the induced IgG demonstrated the capacity to interact with low- and intermediate-affinity FcγRs, suggesting a shared potential for Fc-dependent effector activity irrespective of subclass distribution.

### ChAdOx2-GP38 and ChAdOx2-Gc elicit robust T-cell responses comparable to both ChAdOx2-GPC dose groups, supporting humoural immunity as the main driver of GPC-mediated protection

Given that antibody responses distinguished protected from non-protected groups, we next examined whether T-cell immunity contributed to differences in protection between high- and low-dose ChAdOx2-GPC vaccination while evaluating cellular responses across the subunit constructs. IFN-γ-secreting splenocytes were quantified following stimulation with peptide pools spanning the M segment and mapped to GP38, Gc, and Gn subregions. GP38-specific IFN-γ responses were highest in ChAdOx2-GP38 recipients and, together with standard-dose ChAdOx2-GPC, were significantly elevated compared to GFP controls, with no significant difference between GP38 and GPC groups (GP38 vs GFP, *p* = 0.0002; GPC vs GFP, *p* = 0.0418; Fig. 5A). Similarly, Gc peptide stimulation elicited robust IFN-γ production in ChAdOx2-Gc and standard-dose ChAdOx2-GPC groups, which did not differ significantly from each other but both significantly exceeded GFP controls (Gc vs GFP, *p* = 0.0167; GPC vs GFP, *p* = 0.0045; Fig. 5B). This was reflected in the total summed IFN-γ responses across the M-segment peptide pools, which were also highest in ChAdOx2-Gc and both ChAdOx2-GPC dose groups, all significantly exceeding GFP controls, indicating that the 100-fold reduction in GPC dose did not diminish overall GPC-specific IFN-γ responses (Gc, *p* = 0.0017; GPC LD, *p* = 0.0045; GPC, *p* = 0.0003; Fig. 5C).

**Fig. 5:**
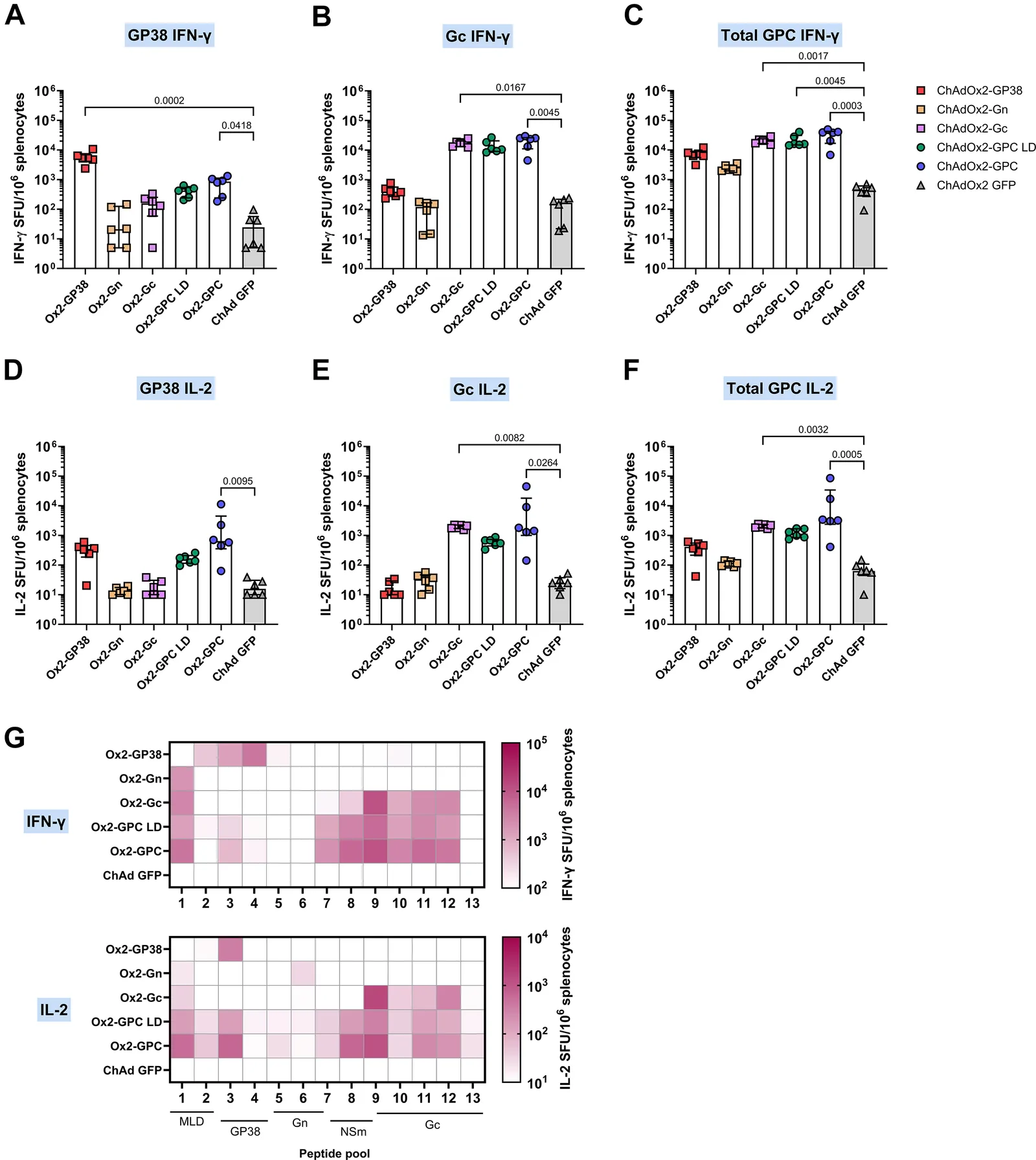
**Robust T-cell immunity is induced by ChAdOx2-GPC, GP38, and Gc vaccines and remains preserved following low-dose GPC vaccination.** IFNAR^−/−^ mice (*n* = 6 per group) were vaccinated with a single dose of ChAdOx2 vectors encoding CCHFV antigens. Twenty-one days post-immunisation, splenocytes were re-stimulated with overlapping peptide pools spanning the CCHFV M segment. **(A**–**C)** IFN-γ-secreting cells were quantified by ELISpot and are shown as summed responses attributed to the (A) GP38, (B) Gc subregions, or **(C)** full GPC. **(D**–**F)** IL-2 secretion was assessed by FluoroSpot for **(D)** GP38, **(E)** Gc, or **(F)** full GPC. **(G)** Heatmap illustrating IFN-γ and IL-2 responses to the 13 individual peptide pools for each vaccine group, with the corresponding five protein domains indicated below. Data represent spot-forming units (SFU) per 10^6^ splenocytes, after initial background subtraction. For dot plots, each symbol represents an individual mouse; bars indicate the median with interquartile range. Statistical analysis was performed using Kruskal–Wallis tests with Dunn's multiple-comparisons test. Exact p-values are shown for statistically significant comparisons. Statistical annotations are shown only for selected biologically relevant comparisons that reached statistical significance; complete statistical results for all comparisons are provided in the Supplementary Table S3.

To further characterise cytokine responses, FluoroSpot assays were performed to quantify IL-2 and TNF-α secretion. GP38-specific IL-2 responses were significantly elevated in ChAdOx2-GPC-vaccinated animals compared to GFP controls (*p* = 0.0095), whereas responses in the ChAdOx2-GP38 subunit group were not significantly different from GFP controls (*p* > 0.05; Fig. 5D). Consistent with the IFN-γ responses, Gc-specific IL-2 responses were significantly higher in standard-dose ChAdOx2-GPC and ChAdOx2-Gc groups than in GFP controls (Gc vs GFP, *p* = 0.0082; GPC vs GFP, *p* = 0.0264; Fig. 5E). Similarly, total GPC-summed IL-2 responses were significantly elevated in both groups compared with GFP controls (Gc vs GFP, *p* = 0.0032; GPC vs GFP, *p* = 0.0005; Fig. 5F). No significant differences in these IL-2 responses were observed between the ChAdOx2-GPC and ChAdOx2-Gc groups (*p* > 0.05).

Mapping of antigen-specific responses showed that IFN-γ and IL-2 responses in Gc-expressing constructs, including ChAdOx2-GPC, were predominantly directed against peptide pools 8–12 corresponding to the Gc region, with a smaller contribution from GP38-associated pools 2–4 in ChAdOx2-GPC, which were primarily targeted in ChAdOx2-GP38-vaccinated animals (Fig. 5G). In contrast, Gn-specific IFN-γ responses were detected only in ChAdOx2-GPC-vaccinated animals and were comparable between standard- and low-dose groups, while no Gn-specific IL-2 responses were detected and the ChAdOx2-Gn subunit vaccine did not elicit responses above GFP control levels (Supplementary Figure S6A). TNF-α responses were low overall and not consistently different from GFP controls (Supplementary Figure S6B and C).

These cellular cytokine data indicate that GP38 and Gc subunit vaccines elicit cellular responses comparable to ChAdOx2-GPC, while T-cell immunity remained intact at the lower ChAdOx2-GPC dose despite loss of protection. Together with the reduced antibody responses observed in the unprotected low-dose ChAdOx2-GPC group, these findings support a primary role for antibody-mediated immunity to GP38 or Gc, rather than T-cell responses, in driving GPC-mediated protection in this model.

### T-cell responses and non-neutralising antigen-specific IgG are induced by ChAdOx2-NP

Having established that single-dose immunisation with ChAdOx2-NP conferred complete survival against lethal CCHFV challenge, we lastly characterised the NP-specific immune responses associated with this efficacy. Sera collected three weeks after vaccination showed uniformly strong NP-specific IgG responses in mice immunised with ChAdOx2-NP, which were significantly higher than those observed in ChAdOx2-GPC- and GFP-immunised animals (both *p* < 0.0001; Fig. 6A). These NP-vaccinated mice showed no measurable IgG reactivity to GP38 or Gc, in contrast to the antigen-specific responses detected in the full-GPC group, consistent with our earlier observations of GPC-induced humoural immunity (Supplementary Figure S7A). Neutralising activity of sera were assessed and shown to be minimal or undetectable in ChAdOx2-NP-immunised mice and similar to levels observed in the full-GPC and GFP control group (Fig. 6B).

**Fig. 6:**
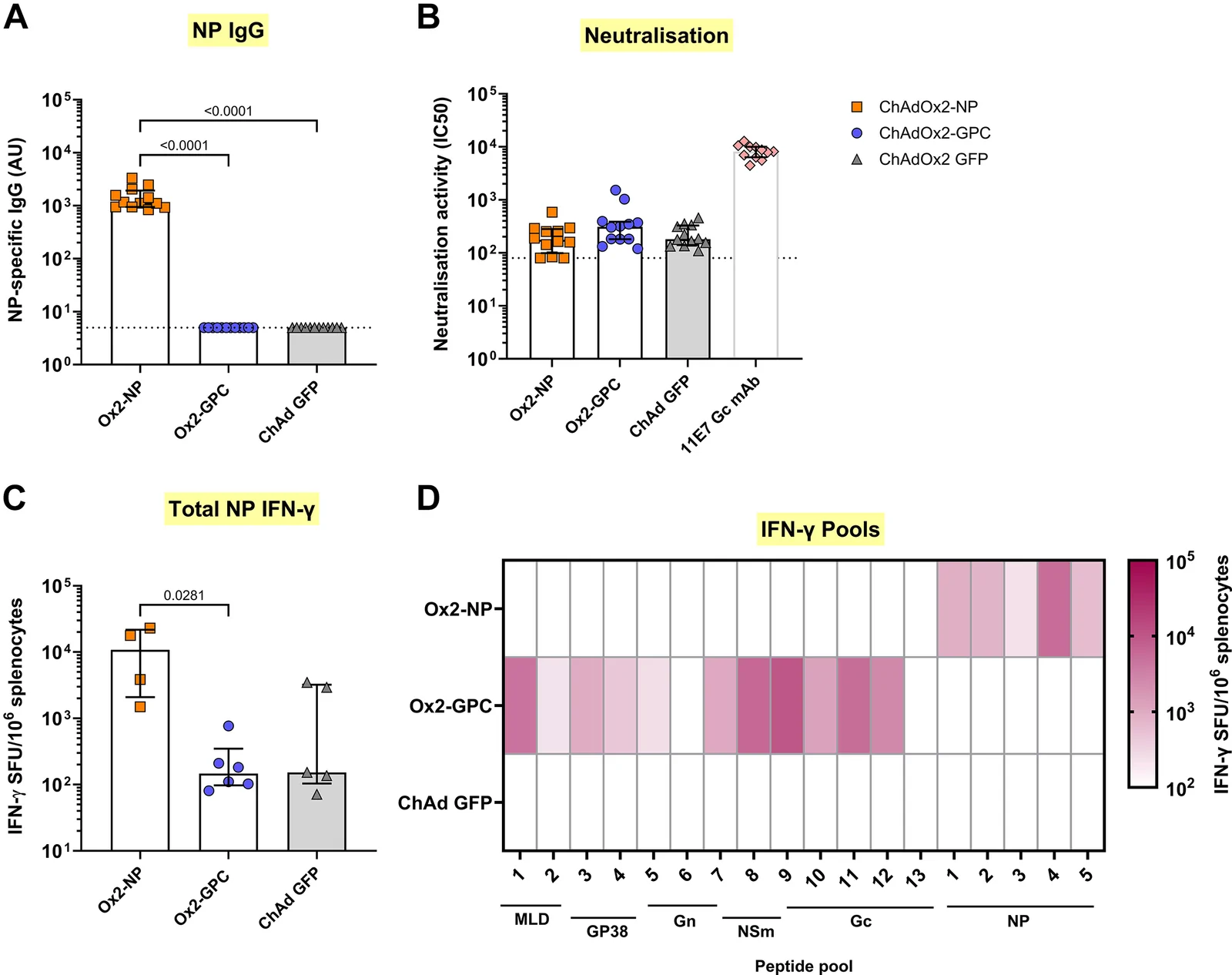
**Single-dose NP immunisation elicits robust NP-specific antibody and T-cell responses.** IFNAR−/− mice (*n* = 12 per group) were vaccinated with a single dose of ChAdOx2 vaccines encoding CCHFV GPC or NP. Three weeks post-immunisation, sera (*n* = 12 per group) and splenocytes (*n* = 6 per group) were collected for analysis. **(A)** NP-specific IgG titres measured by standardised ELISA and expressed as arbitrary units (AU). **(B)** Neutralising antibody activity quantified using the CCHFV tecVLP assay and expressed as IC50 values; the Gc-specific monoclonal antibody 11E7 served as a positive control. **(C)** IFN-γ ELISpot responses shown as total spot-forming units (SFU) per 10^6^ splenocytes following stimulation with peptide pools spanning the CCHFV M or S segments. **(D)** Heatmap illustrating IFN-γ responses to individual M− and S-segment peptide pools across MLD, GP38, Gn, NSm, Gc, and NP regions. Individual data points are presented as scatter plots with medians and interquartile ranges. For ELISpot analyses **(C** and **D)**, *n* = 4 ChAdOx2-NP and *n* = 5 ChAdOx2-GFP animals were available for analysis due to insufficient splenocyte recovery for assay inclusion. Statistical significance was assessed using the Kruskal–Wallis test with Dunn's multiple-comparisons test. Exact p-values are shown for statistically significant comparisons; complete statistical results for all comparisons are provided in the Supplementary Table S4.

To assess cellular immunity, IFN-γ ELISpot assays were performed using peptide pools spanning the CCHFV M and S segments. ChAdOx2-NP immunisation elicited high frequencies of IFN-γ-secreting splenocytes directed toward NP peptides, which were significantly higher than responses observed in ChAdOx2-GPC-immunised animals (*p* = 0.0281), with the strongest responses to peptide pool 4 (Fig. 6C and D). In contrast, ChAdOx2-GPC immunisation elicited responses to multiple GPC-derived pools (Fig. 6D; Supplementary Figure S7B). Together, these data demonstrate that a single dose of ChAdOx2-NP elicits robust NP-specific antibody and T-cell responses in immunocompromised mice, providing a plausible immunological basis for the observed protection.

## Discussion

There remains a critical need for safe and effective human vaccines against CCHFV, especially given the expanding geographic range via *Hyalomma spp.* tick migration. In the present study, we evaluated ChAdOx2-vectored vaccines encoding full-length GPC, individual GPC subunits, or NP. A single dose of ChAdOx2 expressing GP38, Gc, or NP alone conferred complete protection against lethal disease, with GP38 and Gc providing efficacy and immunogenicity comparable to full-length GPC, whereas Gn was weakly immunogenic and provided only limited protection. Notably, a 100-fold decrease in the ChAdOx2-GPC dose markedly diminished protective efficacy despite no reduction in T-cell responses, and in-depth immune analyses indicated a role for non-neutralising antibody-mediated immunity in protection following immunisation with full-length GPC.

Our previous study using ChAdOx2-GPC did not define the contribution of individual GPC components to protection against CCHFV disease, although vaccinated mice showed strong Gc-specific immunity and high GP38-specific IgG responses, the latter not having been reported previously.^21^ Here, we sought to identify the standalone protective components of the M segment by engineering ChAdOx2 constructs expressing Gc, Gn, or GP38 individually, while a ChAdOx2-NP construct was generated to evaluate nucleoprotein-based protection. Initial expression analysis confirmed that all novel ChAdOx2 subunit constructs were expressed as intended: Gn and Gc were correctly processed; GP38 was secreted despite removal of the MLD associated with reduced immunogenicity and processing inefficiencies; and NP was robustly expressed in the cytosol.^15^, ^25^, ^47^ Despite successful expression of all constructs, challenge studies demonstrated that GP38, Gc, and NP were each individually sufficient to confer complete protection in this model, whereas ChAdOx2-Gn was associated with substantially lower survival.

NP has been widely evaluated as a CCHFV vaccine antigen and shown to induce robust immunity, although its protective efficacy has varied by platform and study design.^48^, ^49^, ^50^, ^51^ Recent studies have demonstrated protection in animal models using NP-expressing platforms, including alphavirus-derived replicating RNA (repRNA), mRNA, DNA, viral replicon particles (VRP), and recombinant vesicular stomatitis virus (rVSV), with passive transfer indicating antibody-mediated protection in RNA-based platforms, although the contribution of T-cell responses in other platforms remains incompletely defined.^14^, ^15^, ^17^, ^47^, ^49^, ^52^, ^53^, ^54^, ^55^ In this study, ChAdOx2-NP resulted in 100% survival against lethal challenge despite an absence of detectable neutralising antibodies; NP-specific non-neutralising IgG and robust NP-directed T-cell responses were induced in most animals. The protection associated with NP vaccination here may therefore reflect a combination of antibody functions, including the proposed TRIM21-mediated proteasomal degradation mechanism, and cellular immunity, consistent with recent preclinical reports.^53^, ^56^ These findings reinforce NP's potential as a standalone protective antigen and a valuable component of multivalent vaccine formulations.

By contrast, immunogenicity profiling of ChAdOx2-GPC and the GPC-derived constructs implicated humoural immunity directed towards Gc and GP38 as predominantly associated with protection. The loss of efficacy in the reduced-dose ChAdOx2-GPC group, despite intact T-cell responses, further supports this, demonstrating that cellular immunity alone is insufficient for protection. Only a limited number of studies have experimentally defined GPC-driven correlates of protection using approaches such as immune cell depletion or passive transfer. Among these, a DNA-based GPC vaccine demonstrated CD8^+^ T-cell-dependent protection despite the presence of humoural responses, whereas mechanistic studies of an MVA-vectored GPC vaccine indicated that both humoural and cellular immunity are required.^19^, ^57^ These findings suggest that the relative contribution of immune responses depends on the vaccine platform and the efficiency of GPC antigen expression and presentation. For example, although CCHFV-specific T-cell responses have been implicated in protection, repRNA constructs expressing GPC or Gc elicit strong cellular immunity with limited antibody responses yet confer only partial protection, indicating that T-cell responses alone do not consistently mediate protection across platforms.^16^, ^17^, ^55^ In contrast, many platforms infer correlates based on immunogenicity profiles; DNA-, mRNA-, and rVSV-based approaches achieve high levels of protection when both humoural and cellular responses are induced, although the relative contribution of each arm is rarely defined by mechanistic validation.^15^, ^20^, ^52^, ^54^, ^58^, ^59^ Although not definitively established, humoural immunity to GPC components contributes to protection in rhabdoviral vector-based and engineered glycoprotein approaches, including through non-neutralising mechanisms.^20^, ^23^, ^24^, ^60^, ^61^ In this context, the antibody-driven nature of ChAdOx2-GPC-mediated protection, together with the limited efficacy of T-cell responses alone, highlights the platform-specific influence on immune mechanisms and the challenge of defining definitive correlates of protection across vaccine candidates.

Clinical observations also support a role for humoural immunity, with CCHF patients generating anti-Gc and anti-GP38 antibodies.^62^, ^63^ Neutralising antibodies can arise during infection against the Gc glycoprotein; however, titres are often low or transient and do not consistently associate with survival in animal models or recovery in patients.^18^, ^60^, ^64^, ^65^, ^66^ Consistent with this, we observed limited neutralising activity following GPC immunisation, indicating that protection is likely mediated by non-neutralising mechanisms, although low-level neutralising responses below the assay's detection threshold cannot be excluded. To understand how protection could be achieved in the absence of strong neutralisation, we assessed qualitative features of the antibody response. Subclass and FcγR analyses showed that antigen composition modulated antibody profiles, diverging from the expectation of uniformly Th1-biased responses.^67^, ^68^ ChAdOx2-GP38 induced higher IgG1 titres, consistent with a Th2-skewed profile, whereas ChAdOx2-GPC favoured IgG2a/IgG2b-dominated Th1 responses to both Gc and GP38; ChAdOx2-Gc induced a broadly similar profile with less pronounced Th1 skewing. Despite these differences, GP38- and Gc-specific antibodies exhibited similar FcγR-binding patterns, indicating shared potential for Fc-mediated effector activity. These findings suggest that vaccine-induced antibodies, regardless of subclass profile, may engage similar Fc-dependent mechanisms, including complement pathways, to mediate protection.^69^, ^70^

GP38 has emerged as a key protective antigen within GPC-based vaccine approaches, with vaccine-induced GP38-specific responses correlating with increased survival in animal models and diminished protection upon its removal.^20^, ^24^ Several studies demonstrate that non-neutralising GP38-specific monoclonal antibodies can protect mice from lethal challenge and reverse GP38-induced vascular leakage via complement activation, although Fc-mediated functions cannot be excluded.^20^, ^25^, ^28^, ^71^ Our findings extend this by demonstrating that ChAdOx2-GPC and ChAdOx2-GP38 vaccination elicit GP38-specific non-neutralising polyclonal antibody responses associated with protection, though this remains to be definitively confirmed. By contrast, the non-neutralising effector functions of antibodies targeting the CCHFV Gc glycoprotein are poorly characterised and most Gc-specific antibodies described to date exhibit primarily neutralising activity.^66^, ^72^, ^73^ Future studies will be required to elucidate any non-neutralising effector mechanisms through which these antigens mediate protection, including antibody-dependent cellular cytotoxicity, antibody-dependent phagocytosis, or complement-dependent cytotoxicity following ChAdOx2-GPC or subunit immunisation. Definitive assessment of Fc-mediated mechanisms will also require evaluation in Fcγ receptor-deficient models.

While protection mediated by ChAdOx2 expressing the full-length GPC appears largely attributable to non-neutralising humoural immunity, the standalone GP38 and Gc subunit constructs may achieve protection through more heterogeneous mechanisms, potentially combining non-neutralising and neutralising antibody functions with supportive T-cell responses. Notably, neutralising activity was detected in a subset of ChAdOx2-Gc-immunised mice, but not in ChAdOx2-GPC-immunised animals, consistent with Gc being a recognised neutralisation target and potentially a protective role in this construct. While vaccine-induced neutralising responses have not consistently correlated with protection in animal models, highly potent isolated Gc-specific neutralising antibodies can confer partial protection *in vivo*, supporting a potential contribution in the Gc-only vaccination setting.^65^ However, dose–response studies were not performed for the GP38 and Gc constructs, and therefore the minimal protective dose and potential antibody threshold requirements for these subunit vaccines remain to be defined.

Similarly, although reduced-dose GPC vaccination showed that T-cell responses alone were insufficient for protection, without dose reduction testing we cannot exclude a role for cellular immunity in viral control for animals immunised with ChAdOx2-GP38 or ChAdOx2-Gc. The T-cell responses detected in mice vaccinated with ChAdOx2-GP38 or ChAdOx2-Gc were comparable to or higher to that observed in full GPC-immunised animals, indicating that the ChAdOx2 platform enables efficient processing and presentation of these GPC-derived subunits to T-cells, even when expressed outside the context of the full polyprotein. This contrasts with other GP38-only vaccine attempts with protein- or rhabdoviral-based platforms, which failed to elicit T-cell responses to GP38 when expressed not as part of the GPC.^24^, ^47^ Further delineation of the underlying CD4^+^ and CD8^+^ T-cell subsets will be important to confirm the nature and any protective contribution of these cellular responses across both GPC-based and NP constructs.

Together, these findings inform the rational design of next-generation CCHFV vaccines. Our data support the feasibility of single-dose ChAdOx2-vectored vaccines expressing NP, GPC, or simplified GP38 or Gc constructs as promising candidates for outbreak settings that would suit a CCHFV human immunisation outbreak strategy. This work also provides early evidence that single-dose immunisation with these smaller GP38 and Gc antigens can elicit both humoural and cellular immunity and achieve 100% survival following lethal challenge. However, the considerable genetic diversity of CCHFV raises concerns regarding sequence variability of M segment-encoded vaccine targets such as Gc and especially the less conserved GP38.^72^, ^74^ In our study, GPC and Gc/GP38 subunit antigen constructs demonstrated complete protection against homologous IbAr10200 challenge; in contrast, GPC-based candidates can confer variable efficacy against heterologous strains, including isolates such as Turkey, Oman, or Hoti, with protection ranging from partial to complete depending on the platform and challenge strain used.^17^, ^20^, ^54^, ^59^ The protective efficacy of human-derived Gc- and GP38-specific antibodies have been shown to vary depending on the viral strain for challenge, which may suggest that epitope variability could impact the cross-protective efficacy of ChAdOx2-GP38 and ChAdOx2-Gc by affecting antibody recognition and function.^25^, ^63^, ^66^, ^71^, ^72^ The extent to which ChAdOx2-driven GP38 and Gc immunity translates to broad cross-protection against divergent strains therefore remains to be determined. Looking forward, combining GP38 and Gc, while omitting non-essential regions such as the NSm, may reduce antigenic complexity and help balance non-neutralising and neutralising antibody mechanisms to improve breadth of protection. Moreover, NP remains an attractive, conserved and protective antigen within the ChAdOx2 platform, with some studies reporting greater cross-strain protection than GPC-based vaccines.^15^, ^17^ Combining GPC subunits with NP may maximise the breadth and durability of immune protection, while incorporation of antigens derived from clinically relevant field isolates should be considered to improve cross-lineage protection.^47^

While our study provides important insights into ChAdOx2-induced immunity against CCHFV, there are limitations that warrant consideration. Beyond survival following lethal challenge, demonstrating control of viral replication is an important component of vaccine evaluation. Although viral RNA levels were minimal in protected animals at the study endpoint (day 21 post–challenge), non-protected control animals were euthanised at days 7–8, resulting in non-equivalent temporal sampling. As a result, the current study does not allow assessment of peak viral replication or the extent to which vaccination may limit viral dissemination during acute infection. Future studies incorporating longitudinal sampling and early post–challenge viral quantification will be important to determine how these outcomes relate to control of acute viral replication and viral clearance. Nevertheless, the present findings establish a clear association between protection, reduced pathology, and minimal viral RNA burden at study endpoint. The study was also limited in its ability to assess sex-associated differences in protective efficacy, given the small number of animals of each sex within individual challenge groups in Study I and the use of female animals only in Study II. In addition, variability in survival among vector-only immunised mice was observed in this IFNAR^−/−^ model. While complete lethality was observed in PBS controls in both studies, two GFP-immunised animals survived in Study II, whereas all GFP-immunised animals succumbed under the same conditions in Study I. Sporadic survival in control animals has been reported in interferon receptor-deficient models and is consistent with the known heterogeneity of disease outcome. Importantly, vaccinated groups consistently demonstrated substantial survival benefit and reduced pathology across independent experiments, with clear separation from both PBS and vector-only control groups.

Overall, this work identifies NP, Gc, and GP38 as individually protective antigens against CCHFV. We show that ChAdOx2 is a robust versatile platform for delivery of CCHFV antigens, with non-neutralising mechanisms emerging as contributors to GP38- and Gc-driven protection, and both antibody and T-cell responses contributing to NP-mediated immunity. These findings build on and reinforce pre-clinical and clinical development of ChAdOx2-based CCHFV vaccines, refining understanding of protective immunity and supporting next-generation vaccine design. Future work should test protective candidates in animals with heterologous challenge, and integrate insights from the ongoing Phase I trial of ChAdOx2-GPC to guide clinical translation and pandemic preparedness.

## Contributors

JES, SBR, SD, SCG, and TL conceived and designed the study. SD, EK, SW, SF, SFW, and RH performed the *in vivo* experiments, sample processing and cellular immune assays. IRT and FJS performed and interpreted the pathological analyses. JES, CG and AS designed and generated the ChAdOx2 vectors. JES, FA, SC, and SBR performed ELISA, neutralisation and Luminex assays. JES and SBR analysed and verified the data. JES, CG, KH, SB, DW, SBR, and TL wrote and edited the manuscript. All authors reviewed and approved the final manuscript and accept responsibility for its submission.

## Data sharing statement

All data supporting the findings of this study are included in the main text and Supplementary Material. Additional raw data, relevant research reagents and materials, and the Python code used for PCA analysis will be made available to other researchers for the purposes of reproducing the results or replicating the procedures. Data, code, reagents, and materials can be obtained from the corresponding author upon reasonable request, subject to applicable institutional, intellectual property, material transfer, and third-party requirements.

## Declaration of interests

SCG is named as an inventor on patents covering the ChAdOx1 and ChAdOx2 vaccine technologies and may receive royalties. TL and SBR are inventors and/or contributors to intellectual property licenced by Oxford University Innovation to AstraZeneca. AS is named on a patent application relating to adenoviral vector vaccines filed by Oxford University Innovation Limited. TL was a consultant to Barinthus Biotherapeutics on an unrelated project. TL has received funding outside the submitted work from Seqirus UK Limited, GeoVax Labs Inc, and Infinitopes Limited. All other authors declare no competing interests.
