# Supplementary material for: Immunogenicity and protective efficacy of single-dose adenoviral vaccines expressing Crimean-Congo haemorrhagic fever virus GP38, Gc, or nucleoprotein antigens in mice: a preclinical study
Source: eBioMedicine. 2026 Oct 21;133:106531. doi: 10.1016/j.ebiom.2026.106531 (PMC13716962; doi:10.1016/j.ebiom.2026.106531)
Supplement: Supplementary file 1 [file mmc1.pdf]

Title: Immunogenicity and protective efficacy of single-dose adenoviral vaccines expressing Crimean-Congo haemorrhagic fever virus GP38, Gc, or nucleoprotein antigens in mice: a preclinical study

Co-authors: Jack E. Saunders DPhil[1,3*], Ciaran Gilbride DPhil[1], Alex Sampson DPhil[1,3], Stuart Dowall PhD[2], Emma Kennedy PhD[2], Sarah Webster MSci[2], Susan Fotheringham BSc[2], Stephen Findlay-Wilson PhD[2], Fahad Al-Dainy MSci[1], Susana Camara PhD[1], Khiyam Hussain PhD[1], Ines Ruedas-Torres PhD[2], Francisco J. Salguero PhD[2], Roger Hewson PhD[2], Sarah C. Gilbert PhD[3,4], Sagida Bibi PhD[1], Daniel Wright DPhil[1], Sandra Belij-Rammerstorfer PhD[1], Teresa Lambe PhD[1,3,4]

Supplementary Figures

1) Supplementary Figure 1. Individual animal weight, clinical score, and temperature following CCHFV challenge in Study I.

2) Supplementary Figure 2. Individual animal weight, clinical score, and temperature after CCHFV challenge in Study II.

3) Supplementary Figure 3. Liver and spleen histopathology and viral RNA detection in IFNAR$^{-/-}$ mice immunised with ChAdOx2 vaccines expressing GPC-derived antigens.

4) Supplementary Figure 4. Liver and spleen histopathology and viral RNA detection in IFNAR$^{-/-}$ mice immunised with ChAdOx2-GPC or ChAdOx2-NP.

5) Supplementary Figure 5. Humoral response differences following ChAdOx2 immunisation with GPC subunits.

6) Supplementary Figure 6. IFN-γ ELISpot and multi-cytokine FluoroSpot responses in IFNAR$^{-/-}$ mice 21 days after single-dose GPC-subunit vaccination.

7) Supplementary Figure 7. GPC-specific antibody and T-cell responses following single-dose ChAdOx2-GPC or NP immunisation.

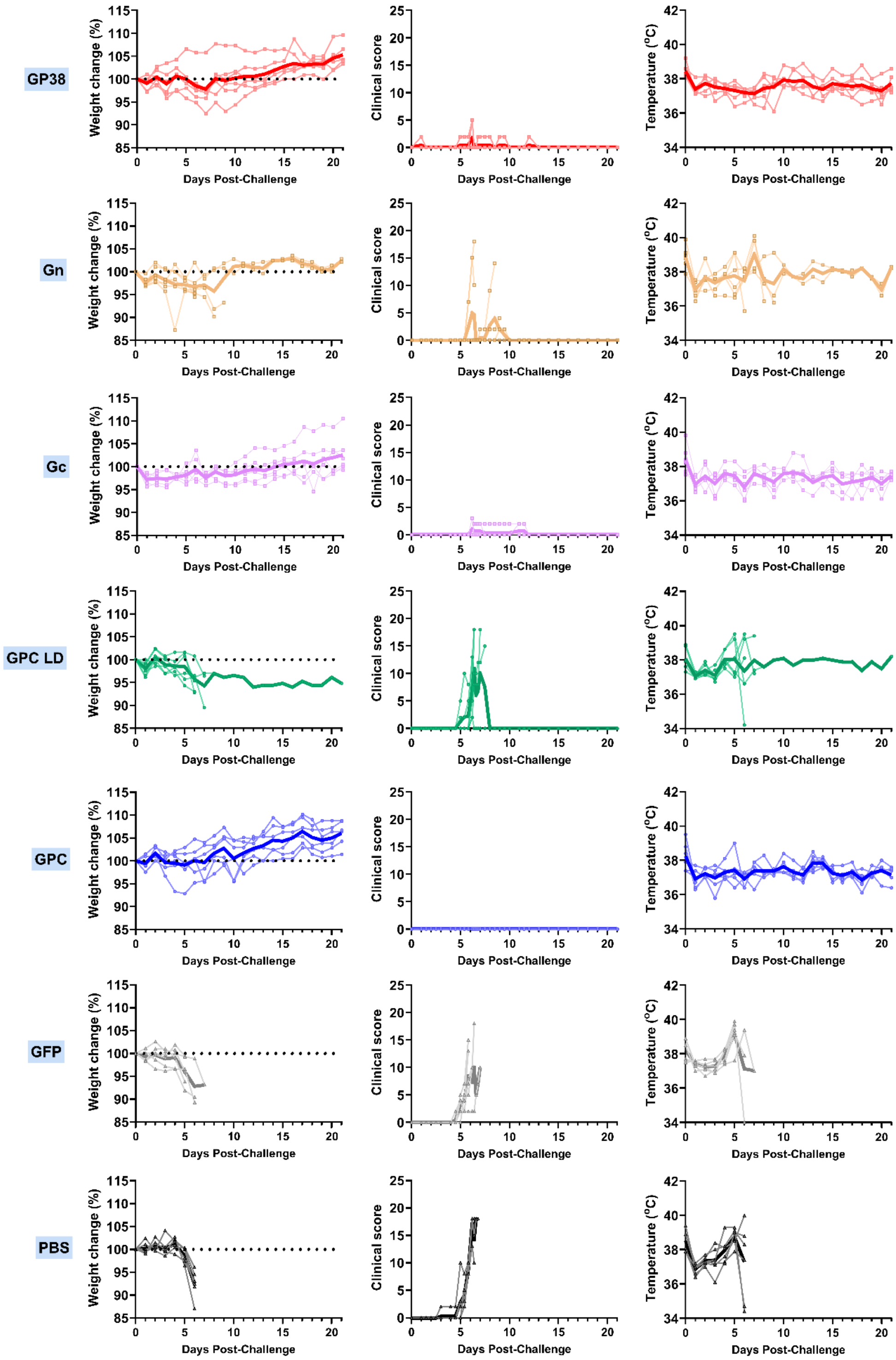

Supplementary Figure 1. Individual animal weight, clinical score, and temperature following CCHFV challenge in Study I. Panels show weight change (% from pre-challenge baseline; left), clinical score at each twice-daily assessment (middle; scoring system described in Methods), and body temperature (°C; right) for A129 IFNAR$^{-/-}$ mice immunised with ChAdOx2-GP38, -Gn, -Gc, -GPC LD (low dose), -GPC (standard dose), ChAdOx2-GFP or PBS controls ($n = 6$ per group). Mice received a single intramuscular dose of 1×10^8 IU (or 1×10^6 IU for the low-dose GPC group) and were challenged three weeks later with CCHFV strain IbAr10200 (day 0). Each line represents an individual animal, with the thicker line indicating the group mean.

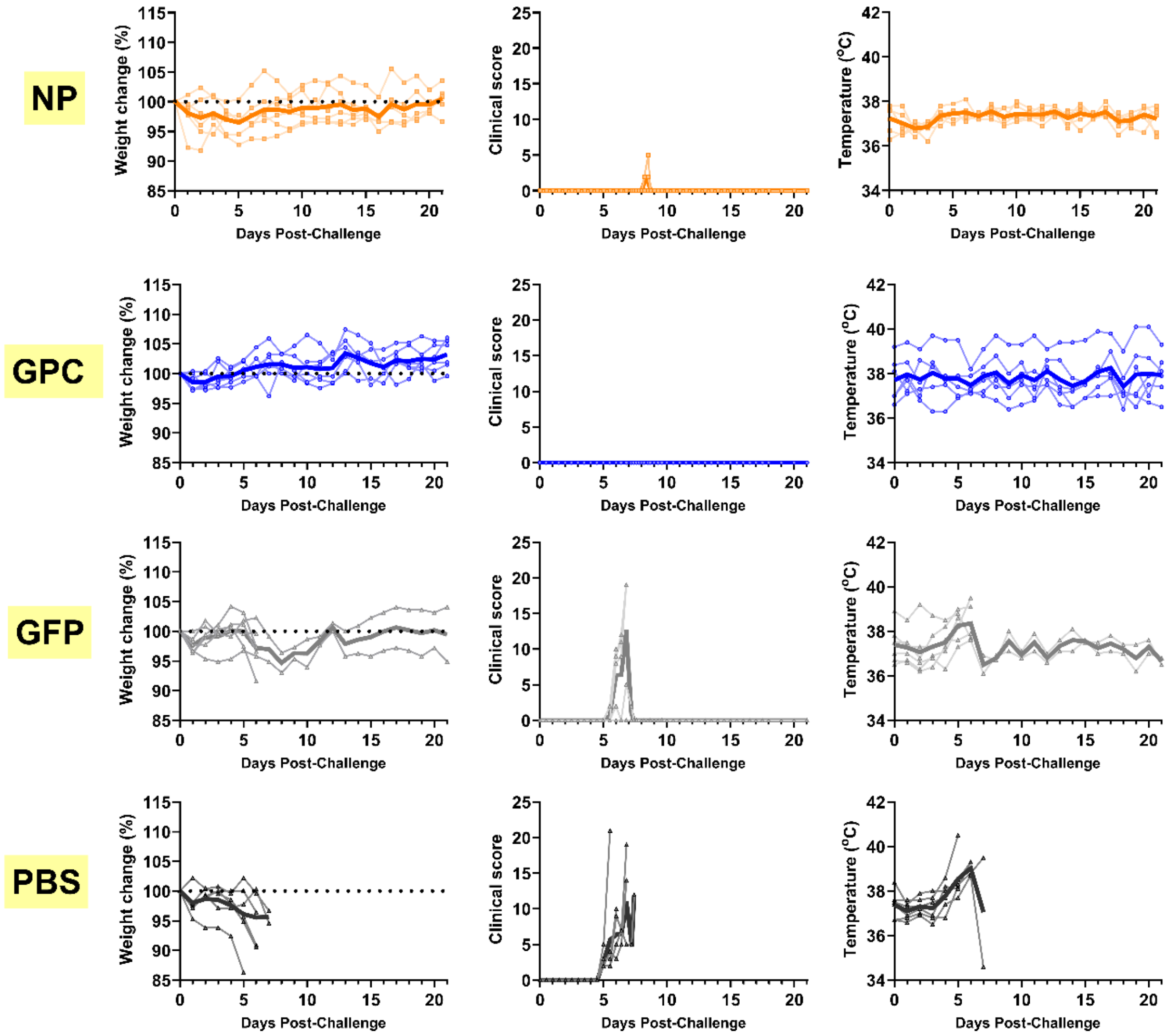

Supplementary Figure 2. Individual animal weight, clinical score, and temperature after CCHFV challenge in Study II. Panels show weight change (% of baseline; left), clinical score at each twice-daily assessment (middle; scoring system described in Methods), and body temperature (°C; right) for A129 IFNAR$^{-/-}$ mice immunised with ChAdOx2-NP, ChAdOx2-GPC, ChAdOx2-GFP, or PBS controls (n = 6 per group). Mice received a single intramuscular dose of 1 × 10^8 IU and were challenged three weeks later with CCHFV strain IbAr10200. Each line represents an individual animal, with the thicker line indicating the group mean.

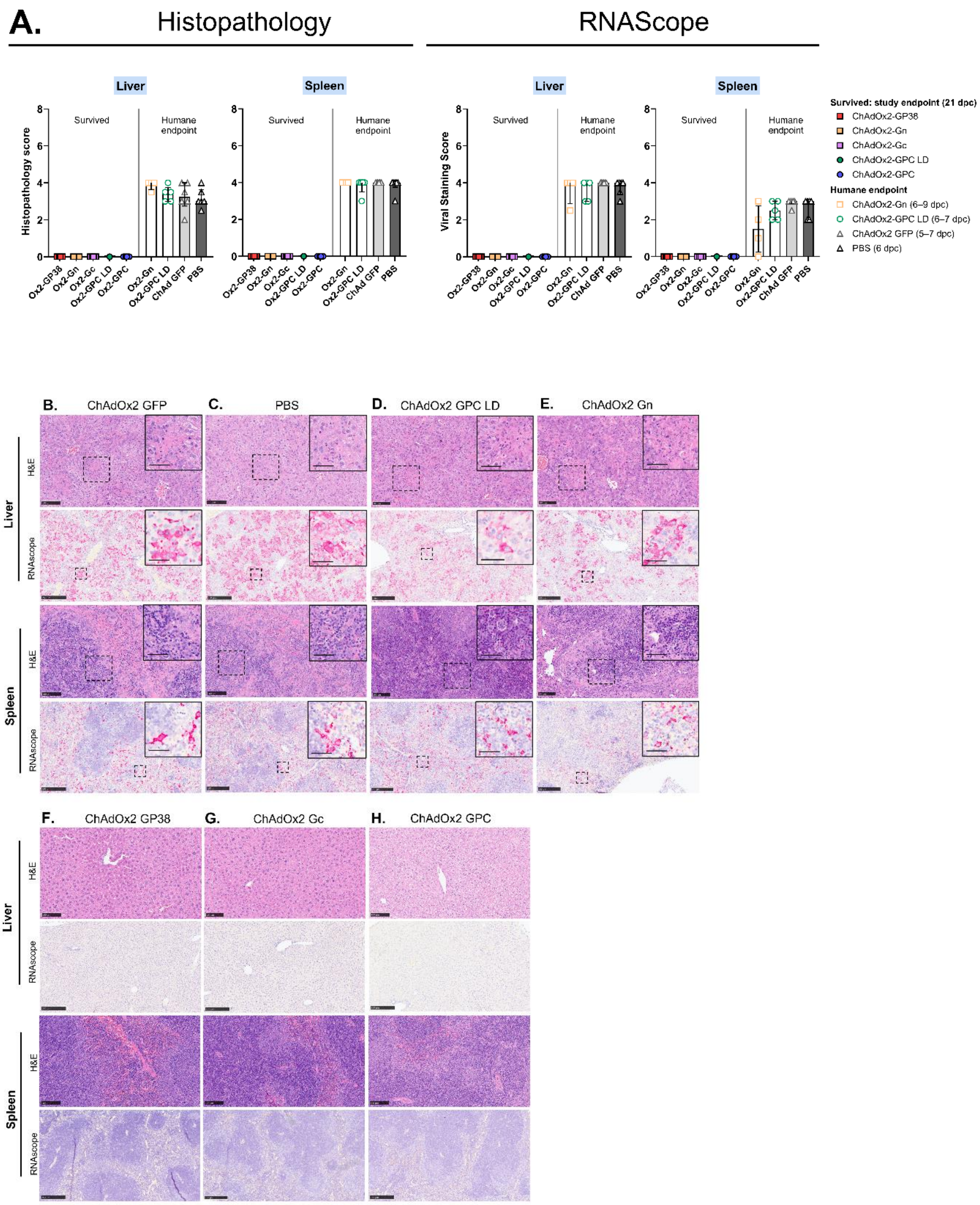

Supplementary Figure 3. Liver and spleen histopathology and viral RNA detection in IFNAR$^{-/-}$ mice immunised with ChAdOx2 vaccines expressing GPC-derived antigens. Tissues were collected from animals that survived to the scheduled study endpoint (21 days post-challenge [dpc]) or upon reaching humane endpoint. **(A)** Tissue histopathology scores (left) and viral RNA staining scores (right) for liver and spleen from mice vaccinated with ChAdOx2-GP38, -Gn, -Gc, or -GPC (standard or low dose), or with ChAdOx2-GFP or PBS controls (n = 6 per group), following lethal challenge with CCHFV strain IbAr10200. Histopathology scoring was performed in a blinded manner using a semi-quantitative 0–4 scale, with the two predefined lesion categories

within each tissue evaluated in combination and assigned a single composite score. Filled symbols represent animals sampled at the study endpoint, whereas open symbols represent animals sampled upon reaching humane endpoint; group-specific humane-endpoint sampling ranges are indicated in the figure key. **(B–E)** Representative Haematoxylin and Eosin (H&E, top row) and RNAscope in situ hybridisation (ISH) for CCHFV RNA (bottom row) in liver and spleen from mice immunised with (B) ChAdOx2-GFP, (C) PBS, (D) low-dose ChAdOx2-GPC, and (E) ChAdOx2-Gn, all showing hepatocellular necrosis, splenic lymphoid depletion, and inflammatory cell infiltration within the red pulp. RNAscope demonstrates abundant viral RNA (red signal). Insets show higher magnification of lesions. **(F–H)** Representative liver and spleen sections from mice immunised with (F) ChAdOx2-GP38, (G) ChAdOx2-Gc, or (H) standard-dose ChAdOx2-GPC, showing normal tissue architecture and absence of detectable viral RNA. Scale bars: H&E, 100 µm; RNAscope, 250 µm; inset images, 50 µm.

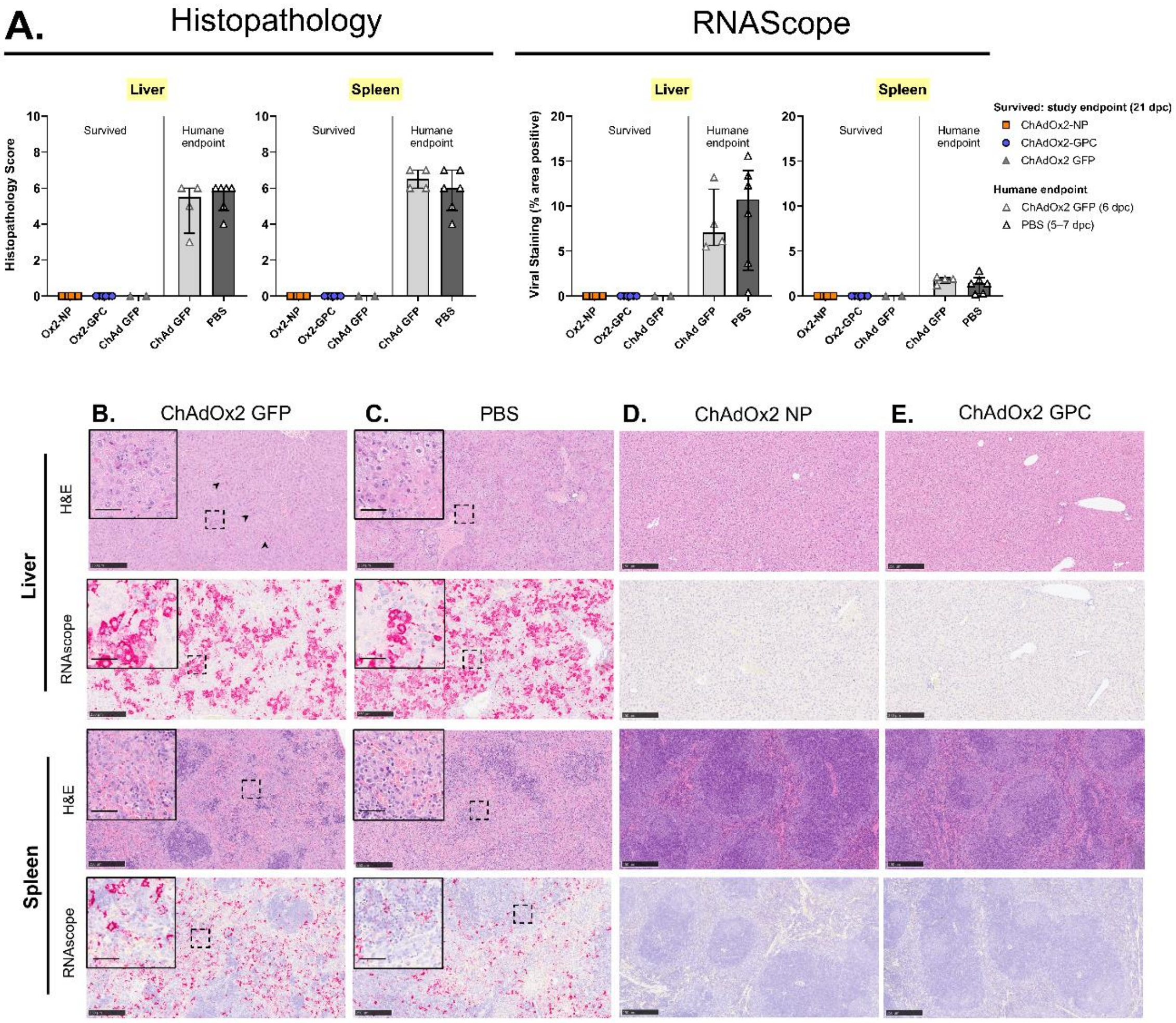

Supplementary Figure 4. Liver and spleen histopathology and viral RNA detection in IFNAR$^{-/-}$ mice immunised with ChAdOx2-GPC or ChAdOx2-NP. Tissues were collected from animals that survived to the scheduled study endpoint (21 days post-challenge [dpc]) or upon reaching humane endpoint. **(A)** Histopathology scores (left) and percentage area positive for viral RNA (right) for liver and spleen from mice vaccinated with ChAdOx2-GPC or ChAdOx2-NP, or ChAdOx2-GFP or PBS controls (*n* = 6 per group), following lethal CCHFV challenge. Histopathology scoring was performed in a blinded manner using a semi-quantitative 0–4 scale, with the two predefined lesion categories within each tissue scored independently and combined to generate the overall histopathology score. Filled symbols represent animals sampled at the study endpoint, whereas open symbols represent animals sampled upon reaching humane endpoint; group-specific humane-endpoint sampling ranges are indicated in the figure key. **(B–C)** Representative H&E (top rows) and RNAscope ISH for CCHFV RNA (bottom rows) in liver and spleen from control animals immunised with (B) ChAdOx2-GFP or (C) PBS, showing hepatocellular necrosis (insets), splenic lymphoid depletion, inflammatory cell infiltration within the red pulp, and abundant viral RNA (red signal). **(D–E)** Representative liver and spleen sections from mice immunised with (D) ChAdOx2-NP or (E) ChAdOx2-GPC, showing preserved architecture and absence of detectable viral RNA by RNAscope. Scale bars: H&E, 100 µm; RNAscope, 250 µm; inset images, 50 µm.

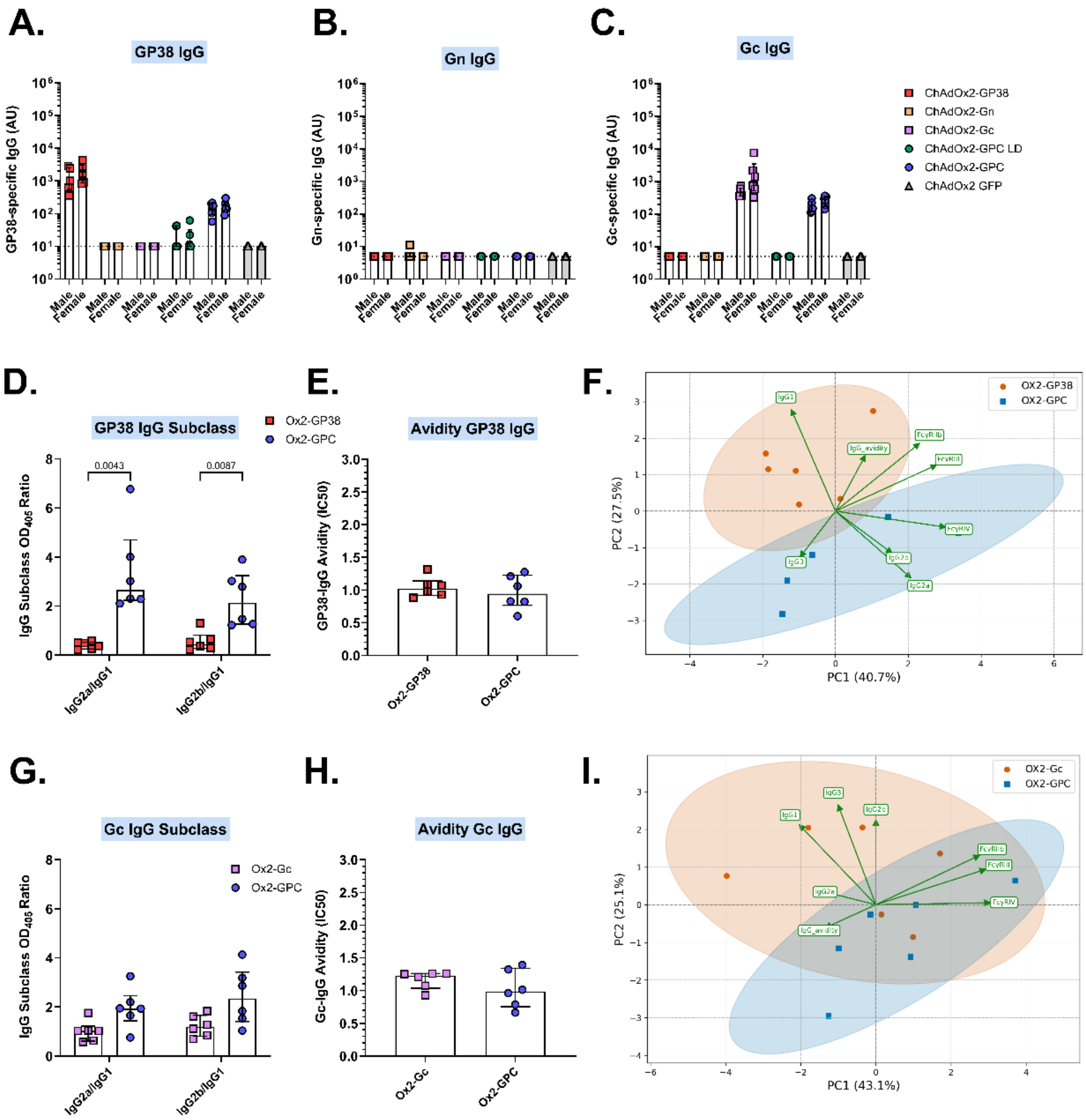

Supplementary Figure 5. Humoral response differences following ChAdOx2 immunisation with GPC subunits. IFNAR$^{-/-}$ mice were vaccinated with a single dose of ChAdOx2 vaccines encoding CCHFV antigens, or with ChAdOx2-GFP (*n* = 12 per group, split equally by sex), and three weeks post-prime mice were either culled for immunogenicity analyses or challenged. Antibody responses were measured in sera collected three weeks post-prime and assayed for **(A)** GP38-specific IgG, **(B)** Gn-specific IgG, and **(C)** Gc-specific IgG. Antibody titres were quantified by standardised ELISA and data are expressed as arbitrary units (AU). **(D–F)** IgG subclass ratios (IgG2a/IgG1 and IgG2b/IgG1) (D), IgG avidity (E), and principal component analysis (PCA) (F) were assessed for GP38-specific antibody responses to visualise differences in humoral response profiles between vaccination groups using sera from surviving challenged animals (*n* = 6 per group). **(G–I)** IgG subclass ratios (G), IgG avidity (H), and PCA (I) were assessed for Gc-specific antibody responses. IgG2/IgG1 ratios were calculated for individual animals as OD_{405} (IgG2a or IgG2b) divided by OD_{405} (IgG1). IgG avidity to GP38 or Gc was determined by treating normalised serum samples with 0–3 M NaSCN followed by IgG ELISA analysis and reported as the concentration of NaSCN required to reduce the OD_{405} to 50% of that measured in the absence of NaSCN (IC_{50}). PCA biplots show clustering of individual animals according to antibody functional profiles, with arrows indicating the contribution (loadings) of individual antibody features to PC1 and PC2, and shaded ellipses representing the 95% confidence regions for each group. For scatter plots, data points represent individual mice, with bars indicating the median and interquartile range. Dotted lines indicate the assay limit of quantification. Statistical significance was assessed using Mann-Whitney tests with Holm-Šídák correction for multiple comparisons, comparing male and female animals within each vaccine group (A–C) or between groups as indicated (D–E, G–H). Exact p-values are shown for statistically significant comparisons; complete statistical results for all comparisons are provided in the **Supplementary Table 5.**

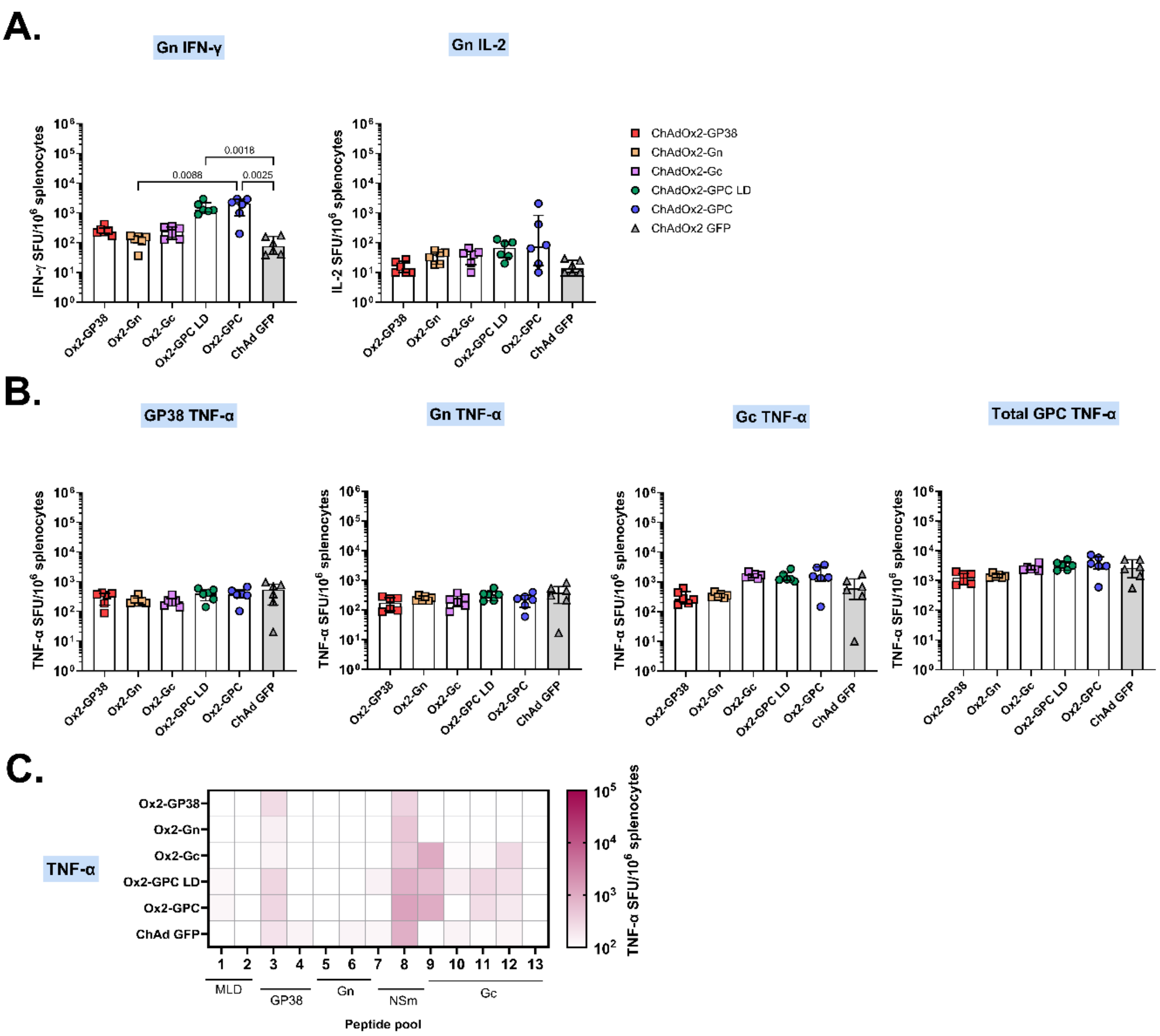

Supplementary Figure 6. IFN-γ ELISpot and multi-cytokine FluoroSpot responses in IFNAR$^{-/-}$ mice 21 days after single-dose GPC-subunit vaccination. Splenocytes were re-stimulated with overlapping peptide pools spanning the CCHFV M segment. IFN-γ-secreting cells were measured by ELISpot, and TNF-α- or IL-2-secreting cells were enumerated by FluoroSpot after background subtraction. Data are expressed as spot-forming units (SFU) per 10^6 splenocytes. **(A)** Gn-specific IFN-γ- and IL-2-secreting cells. **(B)** TNF-α secretion shown as summed responses for GP38, Gn, Gc, or full GPC. **(C)** Heatmap illustrating TNF-α responses to the 13 M segment peptide pools, with corresponding protein domains indicated below. Each symbol represents an individual mouse (*n* = 6 per group); bars indicate the median with the interquartile range. Statistical significance was assessed using a Kruskal-Wallis test with Dunn's multiple-comparisons test. Exact p-values are shown for statistically significant comparisons; complete statistical results for all comparisons are provided in the **Supplementary Table 6.**

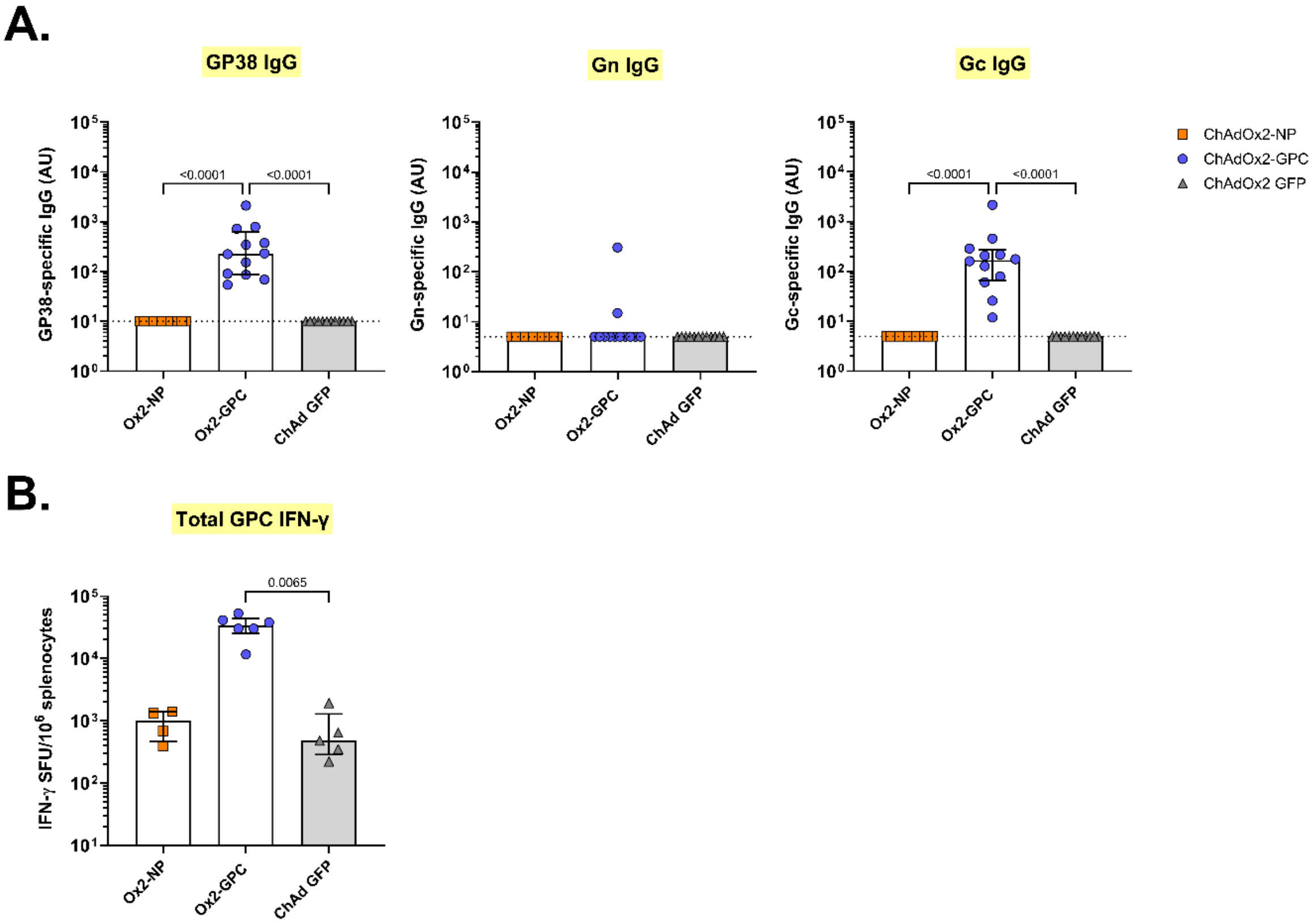

Supplementary Figure 7. GPC-specific antibody and T-cell responses following single-dose ChAdOx2-GPC or NP immunisation. IFNAR$^{-/-}$ mice (*n* = 12 per group) were vaccinated with a single dose of ChAdOx2 vaccines expressing CCHFV GPC or NP. Three weeks post-immunisation, sera (*n* = 12 per group) and splenocytes (*n* = 6 per group) were collected for analysis. **(A)** GP38-, Gn-, and Gc-specific IgG titres measured by standardised ELISAs and expressed as arbitrary units (AU). **(B)** Total IFN-γ ELISpot responses against pooled GPC peptide stimulations shown as spot-forming units (SFU) per 10^6 splenocytes. Data are presented as scatter plots with medians and interquartile ranges. Dotted lines indicate the assay limit of quantification. For ELISpot analyses (B), *n* = 4 ChAdOx2-NP and *n* = 5 ChAdOx2-GFP animals were available for analysis owing to insufficient splenocyte recovery for assay inclusion. Statistical differences between groups were assessed using the Kruskal-Wallis test with Dunn's multiple-comparisons test. Exact p-values are shown for statistically significant comparisons; complete statistical results for all comparisons are provided in the **Supplementary Table 7.**
